# Evolutionary study and phylodynamic pattern of human influenza A/H3N2 virus in Indonesia from 2008 to 2010

**DOI:** 10.1371/journal.pone.0201427

**Published:** 2018-08-01

**Authors:** Agustiningsih Agustiningsih, Hidayat Trimarsanto, Restuadi Restuadi, I. Made Artika, Margaret Hellard, David Handojo Muljono

**Affiliations:** 1 National Institute of Health Research Development, Jakarta, Indonesia; 2 Eijkman Institute for Molecular Biology, Jakarta, Indonesia; 3 Department of Biochemistry, Faculty of Mathematics and Natural Sciences, Bogor Agricultural University, Bogor, Indonesia; 4 Burnet Institute, Melbourne, Australia; 5 Faculty of Medicine, Universitas Hasanuddin, Makassar, Indonesia; 6 Faculty of Medicine and Health, University of Sydney, Sydney, Australia; National Cheng Kung University, TAIWAN

## Abstract

Influenza viruses are by nature unstable with high levels of mutations. The sequential accumulation of mutations in the surface glycoproteins allows the virus to evade the neutralizing antibodies. The consideration of the tropics as the influenza reservoir where viral genetic and antigenic diversity are continually generated and reintroduced into temperate countries makes the study of influenza virus evolution in Indonesia essential. A total of 100 complete coding sequences (CDS) of Hemagglutinin (HA) and Neuraminidase (NA) genes of H3N2 virus were obtained from archived samples of Influenza-Like Illness (ILI) surveillance collected from 2008 to 2010. Our evolutionary and phylogenetic analyses provide insight into the dynamic changes of Indonesian H3N2 virus from 2008 to 2010. Obvious antigenic drift with typical ‘ladder-like’ phylogeny was observed with multiple lineages found in each year, suggesting co-circulation of H3N2 strains at different time periods. The mutational pattern of the Indonesian H3N2 virus was not geographically related as relatively low levels of mutations with similar pattern of relative genetic diversity were observed in various geographical origins. This study reaffirms that the existence of a particular lineage is most likely the result of adaptation or competitive exclusion among different host populations and combination of stochastic ecological factors, rather than its geographical origin alone.

## Introduction

Influenza A viruses cause acute respiratory disease in humans and are responsible for annual epidemic with 3–5 million hospitalization and approximately 500,000 deaths globally [1]. In temperate countries, influenza viruses activities typically occur in the winter season, characterized by the increasing number of influenza cases and associated deaths above a seasonal baseline, known as seasonal influenza pandemic [2]. In contrast, in the tropics, influenza occurs throughout the year without a well-defined pattern, although there is some evidence of higher frequency of respiratory viral infections in rainy season [3].

Two major surface glycoproteins of influenza A viruses—the Haemagglutinin (HA) and Neuraminidase (NA), are the main targets of neutralizing antibodies. Therefore, genes encoding these glycoproteins undergo most frequent mutations. The gradual accumulation of amino acid leads to changes over time within the antigenic sites of HA and NA, referred to as antigenic drift, allowing the virus to escape neutralization by pre-existing antibody [4]. Antigenic drift has contributed to the persistence of epidemics in the community due to inefficient immune clearance by hosts, the occurrence of antiviral resistance [5, 6], implying the need for periodical update of vaccines [7].

Influenza A/H3N2 and Influenza A/H1N1 subtypes together with influenza B have circulated in human population and become the source of annual epidemics in temperate countries. Among those viruses, influenza A/H3N2 subtype has caused frequent outbreaks with associated severe illness [7, 8], and also shows the strongest antigenic drift [4, 9]. The composition of WHO recommended vaccine has been updated several times since 1999 due to recurrent changes of influenza A/H3N2 virus [7].

Indonesia with approximately five million square kilometres of territorial area is the largest country in Southeast Asia. It has more than 17,000 islands and is home to around 250 million people with more than 300 distinct ethnic groups [10, 11]. The bigger western islands of Java, Bali, Sumatra, Kalimantan (Borneo), and Sulawesi are the most populated with faster socio-economic development than the small islands of eastern part of Indonesia. In addition, there is unique ecological and biological arrangement across geographical regions of the country [12]. This diversity is associated with varied resistance and susceptibility to diseases, which could be reflected in the diversity of pathogens due to host-agent interaction among different ethnic populations in Indonesia [13, 14].

The endemicity of poultry H5N1 and high number of human H5N1 cases [15] make Indonesia as an important country associated with the dynamic changes of influenza A viruses. The proximity of domestic animal and human in Indonesia raises concern for the generation of new influenza viruses through possible reassortment of H5N1 with other circulating influenza viruses [16]. The newly generated virus might further obtain beneficial multiple mutations allowing it to adapt and spread among humans. It has been suggested that tropical zone may function as the permanent mixing pool for viruses from around the world [17].

Most of the data concerning seasonal appearance and epidemic of influenza A in human come from temperate countries, and very limited data have been reported on seasonality of influenza in tropical region [18]. With influenza A/H3N2 virus infection occurring throughout the year without well-defined pattern [2], the above situation necessitates studying the dynamics changes and evolution of this virus across years and geographical regions in Indonesia. The present study describes the evolutionary analyses of HA and NA genes of Influenza A/H3N2 virus in Indonesia from 2008 to 2010.

## Materials and methods

### Population study and sampling strategy

The study samples were 242 archived clinical specimens of nasal swabs with confirmed H3N2-positivity obtained during the Influenza-Like Illness (ILI) Surveillance, which was conducted by the National Institute of Health Research and Development (NIHRD), Indonesia, from 2008 to 2010. All ILI samples were collected from outpatients that showed ILI symptoms according to WHO protocols. The confirmation was performed by NIHRD laboratory during the surveillance period following the WHO protocols [19]. All samples were suspended in Hank Balance Salt Solution (HBSS) transport medium and stored properly in -80°C. This study was approved by the Ethics Committee of NIHRD with Federal Wide Assurance (FWA) No. IORG0002751/IRB00003331. All samples were fully anonymized and could not be traced back to individual participants of the study.

### RNA isolation, RT-PCR and direct sequencing

Viral RNA was extracted directly from 140 μL of sample using QIAmp Viral RNA Minikit (Qiagen, Hilden, Germany) according to the manufacturer’s instruction. RT-PCR and direct sequencing to obtain complete coding sequence (CDS) of HA and NA genes were conducted according to previous study [20].

### Dataset preparation

The dataset consisted of sequences generated in this study and sequences retrieved from GenBank that were significant and relevant to be included in the analyses. As illustrated in S1 Fig, the selection of the sequence database was carried out through the following steps:

First, previously published sequences of HA and NA genes of H3N2 from other countries were obtained from GenBank in ‘GenBank-sequence’ format as per July 2, 2013 (10,972 HA and 5,530 NA sequences). The retrieved sequences were then parsed to yield HA-fasta and NA-fasta files containing sequence data with labels indicating isolate accession number, location, and date of isolation as fractioned years.

Second, the sequences from first step were then compared with Indonesian sequences using Usearch, which is a new algorithm for sequence database search that can perform high-scoring of local and global alignment [21]. This process resulted in UC files for HA and NA sequences. The sequences that had 90% similarity were included for the subsequent bioinformatics analysis together with Indonesian samples.

In the third step, the sequences from step two and the Indonesian sequences were aligned using MUSCLE [22]. Maximum likelihood tree of Indonesian and sequences database were constructed by FastTree [23, 24] to find the sequences that are close to Indonesian sequences. Database sequences that did not belong to the same branch as the Indonesian sequences and the non-significance database sequences were removed using patristic analysis [25]. This step resulted in datasets for HA and NA genes that were further analyzed by BEAST and MrBayes methods.

### Phylogenetic analysis, bayesian skyline analysis, TMRCA, and substitution rate estimations

**Fig 1:**
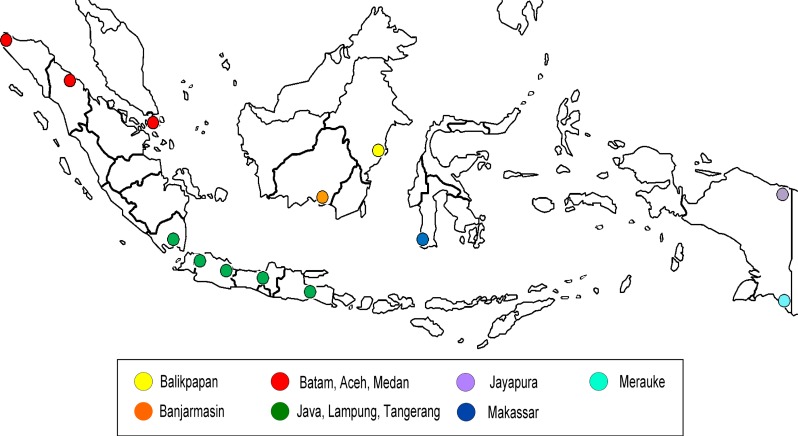
Indonesian map with color-coded geographical origin.

Bayesian Markov Chain Monte Carlo (MCMC) method in BEAST package version 1.6.2 was used to estimate the rate of nucleotide substitutions per site per year, The Most Recent Common Ancestor (TMRCA) and relative genetic diversity (expressed as N_е_τ, where N_e_ is the effective population size and τ denotes the generation time of host-to-host infection) [26]. The general time reversible GTR+I+Г_4_ model of nucleotide substitutions determined by jModelTest prior BEAST analysis [27], relaxed molecular clock model, and Bayesian skyline based on coalescent approach were used for the analysis [28]. We ran MCMC for 100 million generations with sampling every 1000 generations to produce at least 100,000 trees to ensure adequate sample size of the posterior, prior, nucleotide substitution rates and likelihoods (effective sample size >200). The mean substitution rates, TMRCA, and maximum clade credibility (MCC) phylogenetic tree were calculated after 10% removal as burn-in following visual inspection with TRACER version 1.5. The remaining 90.000 sampled trees were summarized using TreeAnnotator to infer the MCC trees, followed by visualization with FigureTree version 1.3.1 where the posterior probability values shown on the major branches [26]. Vaccine strains from 1968 to 2010 and other sequences from Northern Hemisphere, Southern Hemisphere, and tropical countries were included and analysed together with the Indonesian samples. The sequence data were divided into nine taxon groups based on the density and movement of the people and the access for transportation between the respective origins as follows: a) Non Indonesia, b) Indonesia, c) Balikpapan, d) Banjarmasin, e) Batam, Medan and Aceh, f) Java and Lampung, g) Jayapura, h) Makassar, and i) Merauke, as shown in Fig 1.

### Selection analysis

Selection analysis was undertaken using MrBayes version 3.1.2. using a GTR evolution model coupled with gamma rates and codon model, with one million iterations and subsampling of every 100 iterations. The analysis of selective pressure on HA and NA glycoproteins was estimated using MrBayes by looking at the ratio of non-synonymous (*d*N) to synonymous (*d*S) substitutions per site, referred as ω = *d*N/*d*S. The mean probability of 0.5 was set as threshold to determine positive selection pressure [29].

## Results

### Sequence dataset and phylogenetic analysis

One hundred of the 242 samples were successfully sequenced for the CDS of both HA and NA genes. Based on the originating places, the sequences were listed according to the main biogeographical (geographical, ethnological, and zoological) regions of the Indonesian archipelago (Table 1) [12]. The sequences and origins of the samples are shown in S1 Table and have been deposited in Global Initiative on Sharing All Influenza Data (GISAID) with accession number EPI465537-EPI465700, and EPI465712-EPI465748.

**Table 1:** Sample distribution based on biogeographical origins.

Year	Sumatra (Banda Aceh, Batam, Lampung, Medan)	Java	Kalimantan (Banjarmasin, Balikpapan)	East Timor and Bali (Kupang, Denpasar)	Sulawesi (Makassar)	Papua (Merauke, Jayapura)	Total
**2008**	15 (8)	35 (17)	6 (5)	-	34 (12)	21 (9)	111 (51)
**2009**	10 (5)	4 (3)	8 (6)	4 (2)	7 (3)	1 (1)	34 (20)
**2010**	33 (7)	25 (4)	8 (6)	5 (1)	28 (6)	8 (5)	107 (29)
**Total**	58 (20)	64 (24)	22 (17)	9 (3)	69 (21)	30 (15)	252 (100)

Number in bracket represents the number of the sequenced samples

**Fig 2:**
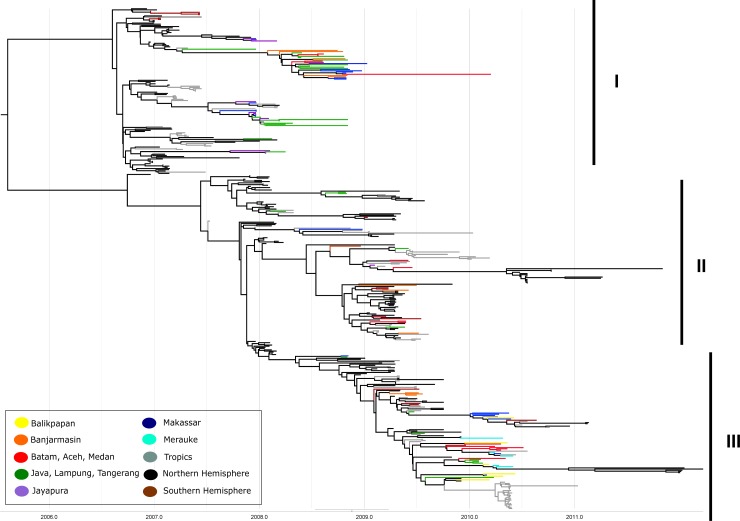
MCC tree from Bayesian analysis of HA gene of H3N2 viruses originated from Indonesia and other countries. Indonesian samples are illustrated in different colored branches according its geographical origin. The numbers I, II and III represent major lineages of the virus. Insert figure is the color-coded geographical origin.

**Fig 3:**
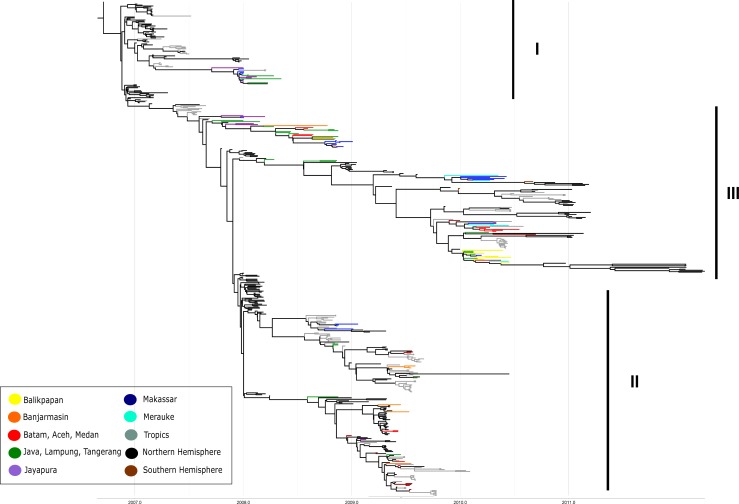
MCC tree from Bayesian analysis of NA gene of H3N2 viruses originated from Indonesia and other countries. Indonesian samples are illustrated in different colored branches according its geographical origin. The numbers I, II and III represent major lineages of the virus. Insert figure is the color-coded geographical origin.

The sequences were analysed together with 320 and 396 sequences (S2 Table), which were selected from 2,364 and 2,495 non-redundant sequences of HA and NA, respectively, of the published sequences in the corresponding years (see Materials and Methods). The phylogenetic tree with maximum clade credibility (MCC) of HA and NA are shown in Figs 2 and 3, respectively. All sequences were grouped into three major lineages: I, II and III, each with multiple sub-lineages. Lineage I consisted of sequences mostly collected in 2007 and 2008 together with the Indonesian sequences of 2008. All strains from this lineage did not continue their lines further than 2008, with the exception of one sample (Batam260). Lineage II mostly consisted of 2009 sequences, while lineage III was composed of 2010 sequences. The topology of the three linages showed the typical ‘ladder-like’ phylogeny with replacement of old strains by newer ones, with each lineage leaving a trunk that became the ancestor of the next generation.

There were two sub-lineages in Lineage III: (a) sub-lineage 1, consisting of two sequences (Makasar917 and Cirebon1828) collected in 2008, and four sequences (Banjarmasin079, Banjarmasin097, Banjarmasin064 and Kupang105) collected in 2009, as well as some from 2010; (b) sub-lineage 2, which mainly consisted of Indonesian sequences from 2010 and one from 2009 (DKI94). Some variants that were distant from the trunk in an evolutionary terminal or dead-end clade were also found.

### Bayesian skyline analysis

**Fig 4:**
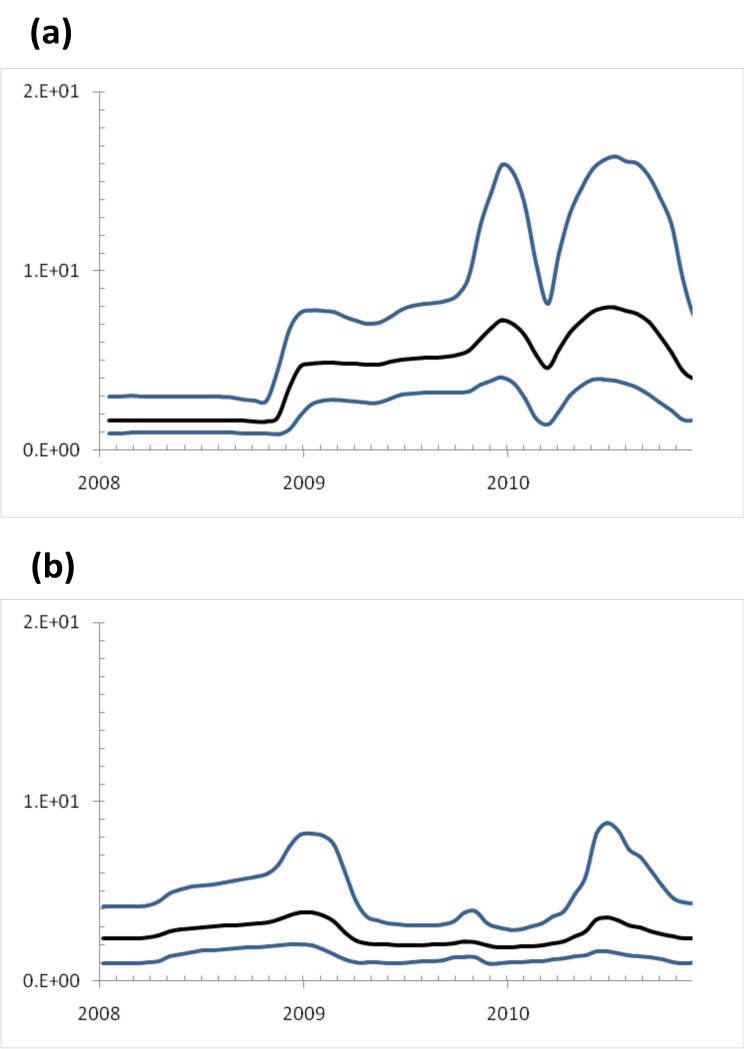
**The Bayesian Skyline plot of HA (A) and (B) NA genes**. The *x*-axis represents the time (mm/yy) and the *y*-axis represents a measure of relative genetic diversity (N_e_t, where the N_e_ is the effective population size and t the generation time from infected host to infected host). Black line represents the mean value while the 95% confidence limits shown in blue line.

The Bayesian skyline plot, which illustrates the changes in genetic diversity through time [30], showed the changing level of genetic diversity of HA and NA genes of Indonesian H3N2 viruses sampled from 2008 to 2010 (Fig 4). There were variations of genetic diversity across years showing three distinct increases with different levels of relative diversity, both in HA and NA genes. The first increase of HA Bayesian skyline plot was at the end of 2008, whereas the second increase was at the end of 2009, followed by rapid decrease at the beginning of 2010. The third increase of genetic diversity occurred from March to June 2010. In NA genes, the first increase of genetic diversity occurred at the end of 2008. The second increase was identified as a small peak from September to October 2009, slightly earlier compared with HA Bayesian skyline plot. The third increase was observed from May to June 2010.

To determine the detailed Bayesian skyline plots according to geographical origin, each sample group was grouped based on the frequencies of people movement within the area and the proximity of one area to the others (S2 Fig). Analysis of population dynamics revealed that the relative genetic diversity both in HA and NA of Indonesian sequences had similar patterns regardless of geographical origin, although Balikpapan and Merauke have slightly different patterns.

### Rates of nucleotide substitution, TMRCA, and selection pressures of the HA and NA genes

The rates of nucleotide substitution and TMRCA are shown in Table 2, while the positively selected sites and *d*_N_/*d*_S_ values in the selection pressure for HA and NA genes are depicted in Table 3. For HA gene, 44 sites were identified to be positively selected. Specifically, 21 positively selected sites were found in HA1 domain, in which eight sites were located at B-cell epitope (antigenic sites A to E), one site at T-cell epitope, two at the receptor binding sites, and one at the glycosylation site, while 23 positively selected sites were found in HA2 domain, with two sites were located at T-cell epitope (S3 Table). For NA gene, 88 positively selected sites were found (S4 Table), with 12 sites were located at B-cell epitope and four sites at T-cell epitope. No positively selected sites were found at the catalytic sites as well as at the framework sites.

**Table 2:** Rates of nucleotide substitution and TMRCA of the HA and NA genes.

Gene	Mean rate of nucleotide substitution (x 10^3^ substitution/per/year)		TMRCA (years)	
	Mean	95% HPD*	Mean	95% HPD*
HA	3.27	2.9–3.7	5.8	5.4–6.3
NA	4.64	4.0–5.4	5.3	5.3–5.4

*HPD: Highest Probability Density

TMRCA: Time of the most recent ancestor.

**Table 3:** Positively selected sites and *d*_N_/*d*_S_ values for HA and NA genes.

Gene		Positively selected sites* (n)			*d*_N_/*d*_S_
		Total	Antigenic sites		
			**B-cell epitope**	**T-cell epitope**	
HA					3.3
	HA1	21	8	1	
	HA2	23		2	
NA		88	12	4	3.0

*using 0.5 of mean probability as threshold

## Discussion

Influenza viruses are unique in that their ecological and evolutionary dynamics occur on the same timescale. Consequently, populations of these fast evolving viruses can accumulate detectable genetic differences in just a few days and can adapt to their environment swiftly [31]. This specific characteristic is examined by investigation of Influenza A/H3N2 virus that continuously changes across different time and location, with a consequence of drift in its phylogeny. To the best of our knowledge, this study is the first in Indonesia that focuses on the evolutionary pattern of the genes encoding the surface glycoproteins influenza A/H3N2 virus across time and geography. In this study, the virus was collected throughout the year with increasing number of samples in rainy seasons (from December to February) when more influenza cases would be more frequent [3]. The ladder-like phylogenies in Figs 3 and 4 represent the antigenic drift of H3N2 as the result of gradual evolution of the virus with multiple mutations occurred cumulatively across years [32]. The central single trunk of each phylogeny characterizes the ancestor of the successful strain with advantageous mutations fixed by natural selection through time [17].

This study observed the presence of positively selected sites which were higher in NA than in HA genes. (Table 3, S3 and S4 Tables). This result was not in accordance with a previous study that reported higher number of positively selected sites in HA compared with those in in NA [33]. However, when analysed more closely, the proportion of positively selected sites found to be in antigenic sites were higher (25%) in HA compared with those observed in NA gene (18%), suggesting that immunological events occurred more frequently in HA than in NA gene. The positively selected sites may vary by the dataset applied, method used, and the significance levels for positive selection determination [18].

Phylogenetic trees both of HA and NA genes revealed that multiple lineages co-circulated and some lineages persisted locally, indicated by the long-line appearance of some strains through the years in the phylogenetic tree. In contrast, the phylogenetic trees also showed that the common ancestor of Indonesian virus existed one or two years before virus sampling (mid-2007). This finding suggests that some lineages became ancestors and were seeded into Northern or Southern Hemisphere in the following seasons or years, and that these viruses might have prevailed and circulated in Indonesian host populations before the dates of collection. Previous study reported that Indonesian H3N2 isolates were detected earlier than the WHO-designated vaccine strain for Northern Hemisphere [34]. This result confirms the long term persistence of virus strains in the tropics, exhibited by co-circulating multiple lineages and often with common ancestors that have prevailed earlier. Consistent with the ‘source-sink’ theory as the evolution model of influenza A virus, the A/H3N2 virus may have persisted locally in the tropics as a reservoir (‘source’ population) before being exported to temperate countries (‘sink’ population) [9].

Phylogenetic analysis also showed a tendency for the viruses isolated from same years to cluster together, regardless geographical origins (Figs 2 and 3); most of the Indonesian samples were intermixed even when the originating islands were separated by oceans. This suggests that these viruses have circulated in various places that are geographically different, with frequent mixing with other viruses from Northern or Southern Hemispheres [35]. The increasing activity of air travel worldwide, with extensive trade and travel between islands or continents connecting millions of people provide a considerable conduit to pathogen transmission. The rapid people movement emphasizes the potential transmission of diseases globally, including the airborne diseases such as influenza [36].

Bayesian skyline plot depicted relatively low genetic diversity within the Indonesian samples through the long and slow seasons in tropical regions [30]. This in contrast with that observed in Northern and Southern Hemispheres with fluctuating levels of relative genetic diversity, which represents stronger natural selection over the short and fast seasons in temperate regions [37]. In relation to neutral evolution, fluctuations in genetic diversity also reflect the underlying changes in the number of infected host [38]. When analysed by geographical origin, a similar pattern of the low genetic diversity of the virus across the Indonesian archipelago was observed, with exception that from Balikpapan and Merauke (S2 Fig). This could be due to the smaller sample size or lower virus population variation in the two places [37].

One limitation of the study was that the collection of samples was carried out during a Ministry of Health geography-based project of three years, which could not enable to study longer period of the antigenic drift. Another limitation was the relatively small number of samples restricted the likelihood of identifying differences between the changes of antigenic sites in each respective year, affecting the ability to interpret significant results. Studies with larger numbers of influenza samples collected over longer time are needed to provide a more distinct evolutionary pattern of Influenza A/H3N2 virus in Indonesia. However, the present study does reveal the evidence of antigenic drift over years and the correspondence of viral strains across geographical regions of the archipelago.

## Conclusions

This study describes the evolutionary pattern of the Influenza A/H3N2 virus in Indonesia from 2008 to 2010 based on the analyses of HA and NA genes. Obvious antigenic drift with typical ‘ladder-like’ phylogeny was observed with multiple lineages found in each year, suggesting co-circulation of H3N2 strains at different time periods despite of the various biogeographical regions in Indonesia. This study reconfirms the long term persistence of virus strains in the tropics before being transferred to temperate regions, suggesting the importance to consistently investigate the characteristics of A/H3N2 strains circulating in the tropics, which may contribute important information regarding further spread of the virus. The existence of a particular lineage is most likely the result of adaptation or competitive exclusion among different host populations and combination of stochastic ecological factors, rather than its geographical origin alone.

## Supporting information

S1 Table List of Indonesian samples and the accession number. (DOCX)

S2 Table List of HA and NA sequences from GenBank database included in evolutionary analyses. (DOCX)

S3 Table Positively selected sites within Indonesian HA gene. (DOCX)

S4 Table Positively selected sited within Indonesian NA gene. (DOCX)

S1 Fig Schematic diagram of bioinformatics analyses. (PDF)

S2 Fig Bayesian skyline plots of HA and NA genes of Indonesian H3N2 virus differentiate according its geographical origin. Datasets were divided based on the samples origin as follow: Balikpapan (a), Banjarmasin (b), Batam, Aceh and Medan (c), Java and Lampung (d), Jayapura (e), Makasar (f), and Merauke (g). The Bayesian skyline plot at the left panel was genererated from HA gene while at the right panel from NA gene. The *x*-axis represents the time (mm/yy) and the *y*-axis represents a measure of relative genetic diveristy (N_e_t, where the N_e_ is the effective population size and t the generation time from infected host to infected host). The black line represents the mean value while the 95% confidence limits shown in blue line.
(PDF)

S3 Fig HA MCC tree with sequence identifier. The Indoneisan sequences were denoted with red labels, while sequences from Southern, Northern Hemispheres, and Tropics were denoted with black labels. Banch color scheme, scale and symbols are similar as those in Fig 2.
(PDF)

S4 Fig NA MCC tree with sequence identifier. The Indoneisan sequences were denoted with red labels, while sequences from Southern, Northern Hemispheres, and Tropics were denoted with black labels. Banch color scheme, scale and symbols are similar as those in Fig 3.
(PDF)

## References

[1] Stohr K. Influenza—WHO cares. Lancet Infect Dis. 2002;2(9):517 Epub 2002/09/11. . 12206966 10.1016/s1473-3099(02)00366-3

[2] Viboud C, Alonso WJ, Simonsen L. Influenza in tropical regions. PLoS Med. 2006;3(4):e89 Epub 2006/03/03. 10.1371/journal.pmed.0030089 . 16509764 PMC1391975

[3] Nelson MI, Simonsen L, Viboud C, Miller MA, Holmes EC. Phylogenetic analysis reveals the global migration of seasonal influenza A viruses. PLoS Pathog. 2007;3(9):1220–8. Epub 2007/10/19. 10.1371/journal.ppat.0030131 ; PubMed Central PMCID: PMC2323296. 17941707 PMC2323296

[4] Grenfell BT, Pybus OG, Gog JR, Wood JL, Daly JM, Mumford JA, et al Unifying the epidemiological and evolutionary dynamics of pathogens. Science. 2004;303(5656):327–32. Epub 2004/01/17. 10.1126/science.1090727 . 14726583

[5] De Jong JC, Rimmelzwaan GF, Fouchier RA, Osterhaus AD. Influenza virus: a master of metamorphosis. J Infect. 2000;40(3):218–28. Epub 2000/07/25. 10.1053/jinf.2000.0652 . 10908015

[6] Deyde VM, Xu X, Bright RA, Shaw M, Smith CB, Zhang Y, et al Surveillance of resistance to adamantanes among influenza A(H3N2) and A(H1N1) viruses isolated worldwide. J Infect Dis. 2007;196(2):249–57. Epub 2007/06/16. 10.1086/518936 . 17570112

[7] Barr IG, McCauley J, Cox N, Daniels R, Engelhardt OG, Fukuda K, et al Epidemiological, antigenic and genetic characteristics of seasonal influenza A(H1N1), A(H3N2) and B influenza viruses: basis for the WHO recommendation on the composition of influenza vaccines for use in the 2009–2010 Northern Hemisphere season. Vaccine. 2010;28(5):1156–67. Epub 2009/12/17. 10.1016/j.vaccine.2009.11.043 . 20004635

[8] Wu P, Goldstein E, Ho LM, Yang L, Nishiura H, Wu JT, et al Excess mortality associated with influenza A and B virus in Hong Kong, 1998–2009. J Infect Dis. 2012;206(12):1862–71. Epub 2012/10/10. 10.1093/infdis/jis628 . 23045622 PMC3502382

[9] Rambaut A, Pybus OG, Nelson MI, Viboud C, Taubenberger JK, Holmes EC. The genomic and epidemiological dynamics of human influenza A virus. Nature. 2008;453(7195):615–9. Epub 2008/04/18. 10.1038/nature06945 . 18418375 PMC2441973

[10] Hugo Pan-Asian SNP Consortium: Abdulla MA, Ahmed I, Assawamakin A, Bhak J, Brahmachari SK, Calacal GC, et al Mapping human genetic diversity in Asia. Science. 2009;326(5959):1541–5. 10.1126/science.1177074 . 20007900

[11] Statistics Indonesia. Statistical Yearbook of Indonesia 2017. Jakarta: BPS-Statistics Indonesia; 2017.

[12] Wallace AR. The Malay Archipelago: Periplus; 1987.

[13] Godlee A, Almond MH, Dong T. Pathogenesis of influenza: virus-host interactions. Expert Rev Anti Infect Ther. 2011;9(8):573–5. 10.1586/eri.11.88 . 21819324

[14] Thedja MD, Muljono DH, Nurainy N, Sukowati CH, Verhoef J, Marzuki S. Ethnogeographical structure of hepatitis B virus genotype distribution in Indonesia and discovery of a new subgenotype, B9. Arch Virol. 2011;156(5):855–68. Epub 2011/02/15. 10.1007/s00705-011-0926-y . 21318309 PMC3081436

[15] Kandun IN, Tresnaningsih E, Purba WH, Lee V, Samaan G, Harun S, et al Factors associated with case fatality of human H5N1 virus infections in Indonesia: a case series. Lancet. 2008;372(9640):744–9. Epub 2008/08/19. 10.1016/S0140-6736(08)61125-3 . 18706688

[16] de Jong MD, Hien TT. Avian influenza A (H5N1). J Clin Virol. 2006;35(1):2–13. Epub 2005/10/11. 10.1016/j.jcv.2005.09.002 . 16213784 PMC7108344

[17] Nelson MI, Holmes EC. The evolution of epidemic influenza. Nat Rev Genet. 2007;8(3):196–205. Epub 2007/01/31. 10.1038/nrg2053 . 17262054

[18] Bragstad K, Nielsen LP, Fomsgaard A. The evolution of human influenza A viruses from 1999 to 2006: a complete genome study. Virology journal. 2008;5:40 Epub 2008/03/08. 10.1186/1743-422X-5-40 ; PubMed Central PMCID: PMC2311284. 18325125 PMC2311284

[19] WHO. WHO Global Influenza Surveillance Network: Manual for the laboratory diagnosis and virological surveillance of influenza2011.

[20] Agustiningsih A, Trimarsanto H, Setiawaty V, Artika IM, Muljono DH. Primer development to obtain complete coding sequence of HA and NA genes of influenza A/H3N2 virus. BMC Res Notes. 2016;9(1):423 Epub 2016/09/01. 10.1186/s13104-016-2235-8 . 27576569 PMC5004302

[21] Edgar RC. Search and clustering orders of magnitude faster than BLAST. Bioinformatics. 2010;26(19):2460–1. Epub 2010/08/17. 10.1093/bioinformatics/btq461 . 20709691

[22] Edgar RC. MUSCLE: multiple sequence alignment with high accuracy and high throughput. Nucleic acids research. 2004;32(5):1792–7. Epub 2004/03/23. 10.1093/nar/gkh340 ; PubMed Central PMCID: PMC390337. 15034147 PMC390337

[23] Price MN, Dehal PS, Arkin AP. FastTree: computing large minimum evolution trees with profiles instead of a distance matrix. Mol Biol Evol. 2009;26(7):1641–50. Epub 2009/04/21. 10.1093/molbev/msp077 ; PubMed Central PMCID: PMC2693737. 19377059 PMC2693737

[24] Price MN, Dehal PS, Arkin AP. FastTree 2—approximately maximum-likelihood trees for large alignments. PLoS One. 2010;5(3):e9490 Epub 2010/03/13. 10.1371/journal.pone.0009490 ; PubMed Central PMCID: PMC2835736. 20224823 PMC2835736

[25] Fourment M, Gibbs MJ. PATRISTIC: a program for calculating patristic distances and graphically comparing the components of genetic change. BMC Evol Biol. 2006;6:1 Epub 2006/01/04. 10.1186/1471-2148-6-1 . 16388682 PMC1352388

[26] Drummond AJ, Rambaut A. BEAST: Bayesian evolutionary analysis by sampling trees. BMC Evol Biol. 2007;7:214 Epub 2007/11/13. 10.1186/1471-2148-7-214 . 17996036 PMC2247476

[27] Posada D. jModelTest: phylogenetic model averaging. Mol Biol Evol. 2008;25(7):1253–6. Epub 2008/04/10. 10.1093/molbev/msn083 . 18397919

[28] Nelson MI, Edelman L, Spiro DJ, Boyne AR, Bera J, Halpin R, et al Molecular epidemiology of A/H3N2 and A/H1N1 influenza virus during a single epidemic season in the United States. PLoS Pathog. 2008;4(8):e1000133 Epub 2008/08/30. 10.1371/journal.ppat.1000133 . 18725925 PMC2495036

[29] Scheffler K, Seoighe C. A Bayesian model comparison approach to inferring positive selection. Mol Biol Evol. 2005;22(12):2531–40. Epub 2005/08/27. 10.1093/molbev/msi250 . 16120799

[30] Drummond AJ, Rambaut A, Shapiro B, Pybus OG. Bayesian coalescent inference of past population dynamics from molecular sequences. Mol Biol Evol. 2005;22(5):1185–92. Epub 2005/02/11. 10.1093/molbev/msi103 . 15703244

[31] Pybus OG, Rambaut A. Evolutionary analysis of the dynamics of viral infectious disease. Nat Rev Genet. 2009;10(8):540–50. Epub 2009/07/01. 10.1038/nrg2583 . 19564871 PMC7097015

[32] Shih AC, Hsiao TC, Ho MS, Li WH. Simultaneous amino acid substitutions at antigenic sites drive influenza A hemagglutinin evolution. Proc Natl Acad Sci U S A. 2007;104(15):6283–8. Epub 2007/03/31. 10.1073/pnas.0701396104 ; PubMed Central PMCID: PMC1851070. 17395716 PMC1851070

[33] Westgeest KB, de Graaf M, Fourment M, Bestebroer TM, van Beek R, Spronken MI, et al Genetic evolution of the neuraminidase of influenza A (H3N2) viruses from 1968 to 2009 and its correspondence to haemagglutinin evolution. J Gen Virol. 2012;93(Pt 9):1996–2007. Epub 2012/06/22. 10.1099/vir.0.043059-0 . 22718569 PMC3542130

[34] Kosasih H, Roselinda Nurhayati, Klimov A, Xiyan X, Lindstrom S, et al Surveillance of Influenza in Indonesia, 2003–2007. Influenza Other Respi Viruses. 2012 Epub 2012/07/19. 10.1111/j.1750-2659.2012.00403.x . 22804910 PMC5779827

[35] Russell CA, Jones TC, Barr IG, Cox NJ, Garten RJ, Gregory V, et al The global circulation of seasonal influenza A (H3N2) viruses. Science. 2008;320(5874):340–6. Epub 2008/04/19. 10.1126/science.1154137 . 18420927

[36] Colizza V, Barrat A, Barthelemy M, Vespignani A. The role of the airline transportation network in the prediction and predictability of global epidemics. Proc Natl Acad Sci U S A. 2006;103(7):2015–20. Epub 2006/02/08. 10.1073/pnas.0510525103 . 16461461 PMC1413717

[37] Bahl J, Nelson MI, Chan KH, Chen R, Vijaykrishna D, Halpin RA, et al Temporally structured metapopulation dynamics and persistence of influenza A H3N2 virus in humans. Proc Natl Acad Sci U S A. 2011;108(48):19359–64. Epub 2011/11/16. 10.1073/pnas.1109314108 ; PubMed Central PMCID: PMC3228450. 22084096 PMC3228450

[38] Holmes EC, Grenfell BT. Discovering the phylodynamics of RNA viruses. PLoS Comput Biol. 2009;5(10):e1000505 Epub 2009/10/27. 10.1371/journal.pcbi.1000505 . 19855824 PMC2756585

