# Supplementary material for: Evolutionary study and phylodynamic pattern of human influenza A/H3N2 virus in Indonesia from 2008 to 2010
Source: PLoS One. 2018 Aug 1;13(8):e0201427. doi: 10.1371/journal.pone.0201427 (PMC6070282; doi:10.1371/journal.pone.0201427)
Supplement: S1 Table — (DOCX) [file pone.0201427.s001.docx]

**S1 Table. List of Indonesian samples and the accession number**

| **Place** | **HA gene** | **NA gene** | **Collection date** |
| --- | --- | --- | --- |
| Banjarmasin | EPI465537 \| A/Indonesia/NIHRDI-BM705/2008 | EPI465538 \| A/Indonesia/NIHRDI-BM705/2008 | 31/10/2008 |
|  | EPI465539 \| A/Indonesia/NIHRDI-BM707/2008 | EPI465540 \| A/Indonesia/NIHRDI-BM707/2008 | 31/10/2008 |
|  | EPI465541 \| A/Indonesia/NIHRDI-BM711/2008 | EPI465542 \| A/Indonesia/NIHRDI-BM711/2008 | 08/11/2008 |
|  | EPI465543 \| A/Indonesia/NIHRDI-BM712/2008 | EPI465544 \| A/Indonesia/NIHRDI-BM712/2008 | 10/11/2008 |
|  | EPI465545 \| A/Indonesia/NIHRDI-BM714/2008 | EPI465546 \| A/Indonesia/NIHRDI-BM714/2008 | 15/11/2008 |
|  | EPI465642 \| A/Influenza/NIHRDI-BM44/2009 | EPI465643 \| A/Influenza/NIHRDI-BM44/2009 | 16/06/2009 |
|  | EPI465644 \| A/Indonesia/NIHRDI-BM64/2009 | EPI465645 \| A/Indonesia/NIHRDI-BM64/2009 | 14/07/2009 |
|  | EPI465646 \| A/Indonesia/NIHRDI-BM66/2009 | EPI465647 \| A/Indonesia/NIHRDI-BM66/2009 | 14/07/2009 |
|  | EPI465648 \| A/Indonesia/NIHRDI-BM78/2009 | EPI465649 \| A/Indonesia/NIHRDI-BM78/2009 | 21/07/2009 |
|  | EPI465650 \| A/Indonesia/NIHRDI-BM79/2009 | EPI465651 \| A/Indonesia/NIHRDI-BM79/2009 | 21/07/2009 |
|  | EPI465652 \| A/Indonesia/NIHRDI-BM97/2009 | EPI465653 \| A/Indonesia/NIHRDI-BM97/2009 | 04/08/2009 |
| Balikapapan | EPI465680 \| A/Indonesia/NIHRDI-BP91/2010 | EPI465681 \| A/Indonesia/NIHRDI-BP91/2010 | 23/03/2010 |
|  | EPI465682 \| A/Indonesia/NIHRDI-BP103/2010 | EPI465683 \| A/Indonesia/NIHRDI-BP103/2010 | 24/03/2010 |
|  | EPI465684 \| A/Indonesia/NIHRDI-BP107/2010 | EPI465685 \| A/Indonesia/NIHRDI-BP107/2010 | 10/05/2010 |
|  | EPI465686 \| A/Indonesia/NIHRDI-BP109/2010 | EPI465687 \| A/Indonesia/NIHRDI-BP109/2010 | 26/05/2010 |
|  | EPI465688 \| A/Indonesia/NIHRDI-BP112/2010 | EPI465689 \| A/Indonesia/NIHRDI-BP112/2010 | 22/06/2010 |
|  | EPI465690 \| A/Indonesia/NIHRDI-BP116/2010 | EPI465691 \| A/Indonesia/NIHRDI-BP116/2010 | 17/06/2010 |
| Batam, Aceh, Medan | EPI465547 \| A/Indonesia/NIHRDI-BT163/2008 | EPI465548 \| A/Indonesia/NIHRDI-BT163/2008 | 07/08/2008 |
|  | EPI465549 \| A/Indonesia/NIHRDI-BT188/2008 | EPI465550 \| A/Indonesia/NIHRDI-BT188/2008 | 21/08/2008 |
|  | EPI465551 \| A/Indonesia/NIHRDI-BT194/2008 | EPI465552 \| A/Indonesia/NIHRDI-BT194/2008 | 25/08/2008 |
|  | EPI465553 \| A/Indonesia/NIHRDI-BT195/2008 | EPI465554 \| A/Indonesia/NIHRDI-BT195/2008 | 25/08/2008 |
|  | EPI465654 \| A/Indonesia/NIHRDI-BT57/2009 | EPI465655 \| A/Indonesia/NIHRDI-BT57/2009 | 15/06/2009 |
|  | EPI465656 \| A/Indonesia/NIHRDI-BT62/2009 | EPI465657 \| A/Indonesia/NIHRDI-BT62/2009 | 15/06/2009 |
|  | EPI465658 \| A/Indonesia/NIHRDI-BT77/2009 | EPI465659 \| A/Indonesia/NIHRDI-BT77/2009 | 29/06/2009 |
|  | EPI465660 \| A/Indonesia/NIHRDI-BT87/2009 | EPI465661 \| A/Indonesia/NIHRDI-BT87/2009 | 30/06/2009 |
|  | EPI465662 \| A/Indonesia/NIHRDI-BT226/2009 | EPI465663 \| A/Indonesia/NIHRDI-BT226/2009 | 30/03/2009 |
|  | EPI465692 \| A/Indonesia/NIHRDI-BTM260/2010 | EPI465693 \| A/Indonesia/NIHRDI-BTM260/2010 | 29/03/2010 |
|  | EPI465694 \| A/Indonesia/NIHRDI-BTM272/2010 | EPI465695 \| A/Indonesia/NIHRDI-BTM272/2010 | 12/04/2010 |
|  | EPI465696 \| A/Indonesia/NIHRDI-BTM290/2010 | EPI465697 \| A/Indonesia/NIHRDI-BTM290/2010 | 03/05/2010 |
|  | EPI465700 \| A/Indonesia/NIHRDI-MDN190/2010 | EPI465712 \| A/Indonesia/NIHRDI-MDN190/2010 | 19/05/2010 |
|  | EPI465735 \| A/Indonesia/NIHRDI-NAD182/2010 | EPI465736 \| A/Indonesia/NIHRDI-NAD182/2010 | 03/05/2010 |
|  | EPI465737 \| A/Indonesia/NIHRDI-NAD193/2010 | EPI465738 \| A/Indonesia/NIHRDI-NAD193/2010 | 25/05/2010 |
|  | EPI465739 \| A/Indonesia/NIHRDI-NAD216/2010 | EPI465740 \| A/Indonesia/NIHRDI-NAD216/2010 | 20/07/2010 |
| Java, Lampung | EPI465555 \| A/Indonesia/NIHRDI-JB1427/2008 | EPI465556 \| A/Indonesia/NIHRDI-JB1427/2008 | 27/02/2008 |
|  | EPI465557 \| A/Indonesia/NIHRDI-JB1828/2008 | EPI465558 \| A/Indonesia/NIHRDI-JB1828/2008 | 15/11/2008 |
|  | EPI465559 \| A/Indonesia/NIHRDI-JK538/2008 | EPI465560 \| A/Indonesia/NIHRDI-JK538/2008 | 02/01/2008 |
|  | EPI465561 \| A/Indonesia/NIHRDI-JK661/2008 | EPI465562 \| A/Indonesia/NIHRDI-JK661/2008 | 25/03/2008 |
|  | EPI465563 \| A/Indonesia/NIHRDI-JK687/2008 | EPI465564 \| A/Indonesia/NIHRDI-JK687/2008 | 15/04/2008 |
|  | EPI465565 \| A/Indonesia/NIHRDI-JK770/2008 | EPI465566 \| A/Indonesia/NIHRDI-JK770/2008 | 07/07/2008 |
|  | EPI465567 \| A/Indonesia/NIHRDI-JK832/2008 | EPI465568 \| A/Indonesia/NIHRDI-JK832/2008 | 13/11/2008 |
|  | EPI465569 \| A/Indonesia/NIHRDI-LP516/2008 | EPI465570 \| A/Indonesia/NIHRDI-LP516/2008 | 08/06/2008 |
|  | EPI465571 \| A/Indonesia/NIHRDI-LP518/2008 | EPI465572 \| A/Indonesia/NIHRDI-LP518/2008 | 10/06/2008 |
|  | EPI465573 \| A/Indonesia/NIHRDI-LP576/2008 | EPI465574 \| A/Indonesia/NIHRDI-LP576/2008 | 04/11/2008 |
|  | EPI465575 \| A/Indonesia/NIHRDI-LP580/2008 | EPI465576 \| A/Indonesia/NIHRDI-LP580/2008 | 10/11/2008 |
|  | EPI465577 \| A/Indonesia/NIHRDI-ML696/2008 | EPI465578 \| A/Indonesia/NIHRDI-ML696/2008 | 19/01/2008 |
|  | EPI465579 \| A/Indonesia/NIHRDI-ML746/2008 | EPI465580 \| A/Indonesia/NIHRDI-ML746/2008 | 14/04/2008 |
|  | EPI465581 \| A/Indonesia/NIHRDI-ML750/2008 | EPI465582 \| A/Indonesia/NIHRDI-ML750/2008 | 14/04/2008 |
|  | EPI465583 \| A/Indonesia/NIHRDI-ML769/2008 | EPI465584 \| A/Indonesia/NIHRDI-ML769/2008 | 09/05/2008 |
|  | EPI465627 \| A/Indonesia/NIHRDI-SL925/2008 | EPI465628 \| A/Indonesia/NIHRDI-SL925/2008 | 04/11/2008 |
|  | EPI465629 \| A/Indonesia/NIHRDI-SL935/2008 | EPI465630 \| A/Indonesia/NIHRDI-SL935/2008 | 17/11/2008 |
|  | EPI465631 \| A/Indonesia/NIHRDI-SL937/2008 | EPI465633 \| A/Indonesia/NIHRDI-SL937/2008 | 17/11/2008 |
|  | EPI465634 \| A/Indonesia/NIHRDI-SL938/2008 | EPI465635 \| A/Indonesia/NIHRDI-SL938/2008 | 17/11/2008 |
|  | EPI465636 \| A/Indonesia/NIHRDI-SL939/2008 | EPI465637 \| A/Indonesia/NIHRDI-SL939/2008 | 17/11/2008 |
|  | EPI465638 \| A/Indonesia/NIHRDI-SL940/2008 | EPI465639 \| A/Indonesia/NIHRDI-SL940/2008 | 17/11/2008 |
|  | EPI465640 \| A/Indonesia/NIHRDI-BL58/2009 | EPI465641 \| A/Indonesia/NIHRDI-BL58/2009 | 03/06/2009 |
|  | EPI465664 \| A/Indonesia/NIHRDI-DKI54/2009 | EPI465665 \| A/Indonesia/NIHRDI-DKI54/2009 | 16/06/2009 |
|  | EPI465666 \| A/Indonesia/NIHRDI-DKI94/2009 | EPI465667 \| A/Indonesia/NIHRDI-DKI94/2009 | 11/08/2009 |
|  | EPI465668 \| A/Indonesia/NIHRDI-KP105/2009 | EPI465669 \| A/Indonesia/NIHRDI-KP105/2009 | 19/08/2009 |
|  | EPI465678 \| A/Indonesia/NIHRDI-TG077/2009 | EPI465679 \| A/Indonesia/NIHRDI-TG077/2009 | 06/07/2009 |
|  | EPI465698 \| A/Indonesia/NIHRDI-DPS236/2010 | EPI465699 \| A/Indonesia/NIHRDI-DPS236/2010 | 08/04/2010 |
|  | EPI465741 \| A/Indonesia/NIHRDI-SMG205/2010 | EPI465742 \| A/Indonesia/NIHRDI-SMG205/2010 | 15/02/2010 |
|  | EPI465743 \| A/Indonesia/NIHRDI-SMG208/2010 | EPI465744 \| A/Indonesia/NIHRDI-SMG208/2010 | 15/02/2010 |
|  | EPI465745 \| A/Indonesia/NIHRDI-SMG213/2010 | EPI465746 \| A/Indonesia/NIHRDI-SMG213/2010 | 22/02/2010 |
|  | EPI465747 \| A/Indonesia/NIHRDI-SMG219/2010 | EPI465748 \| A/Indonesia/NIHRDI-SMG219/2010 | 01/03/2010 |
| Jayapura | EPI465609 \| A/Indonesia/NIHRDI-PP179/2008 | EPI465610 \| A/Indonesia/NIHRDI-PP179/2008 | 02/01/2008 |
|  | EPI465611 \| A/Indonesia/NIHRDI-PP180/2008 | EPI465612 \| A/Indonesia/NIHRDI-PP180/2008 | 02/01/2008 |
|  | EPI465613 \| A/Indonesia/NIHRDI-PP185/2008 | EPI465614 \| A/Indonesia/NIHRDI-PP185/2008 | 02/01/2008 |
|  | EPI465615 \| A/Indonesia/NIHRDI-PP188/2008 | EPI465616 \| A/Indonesia/NIHRDI-PP188/2008 | 02/01/2008 |
|  | EPI465617 \| A/Indonesia/NIHRDI-PP219/2008 | EPI465618 \| A/Indonesia/NIHRDI-PP219/2008 | 30/01/2008 |
|  | EPI465619 \| A/Indonesia/NIHRDI-PP226/2008 | EPI465620 \| A/Indonesia/NIHRDI-PP226/2008 | 06/02/2008 |
|  | EPI465621 \| A/Indonesia/NIHRDI-PP232/2008 | EPI465622 \| A/Indonesia/NIHRDI-PP232/2008 | 16/02/2008 |
|  | EPI465623 \| A/Indonesia/NIHRDI-PP235/2008 | EPI465624 \| A/Indonesia/NIHRDI-PP235/2008 | 20/02/2008 |
|  | EPI465625 \| A/Indonesia/NIHRDI-PP255/2008 | EPI465626 \| A/Indonesia/NIHRDI-PP255/2008 | 15/03/2008 |
|  | EPI465676 \| A/Indonesia/NIHRDI-PP413/2009 | EPI465677 \| A/Indonesia/NIHRDI-PP413/2009 | 19/02/2009 |
| Makassar | EPI465585 \| A/Indonesia/NIHRDI-MS618/2008 | EPI465586 \| A/Indonesia/NIHRDI-MS618/2008 | 02/01/2008 |
|  | EPI465587 \| A/Indonesia/NIHRDI-MS623/2008 | EPI465588 \| A/Indonesia/NIHRDI-MS623/2008 | 03/01/2008 |
|  | EPI465589 \| A/Indonesia/NIHRDI-MS633/2008 | EPI465590 \| A/Indonesia/NIHRDI-MS633/2008 | 04/01/2008 |
|  | EPI465591 \| A/Indonesia/NIHRDI-MS634/2008 | EPI465592 \| A/Indonesia/NIHRDI-MS634/2008 | 04/01/2008 |
|  | EPI465593 \| A/Indonesia/NIHRDI-MS635/2008 | EPI465594 \| A/Indonesia/NIHRDI-MS635/2008 | 04/01/2008 |
|  | EPI465595 \| A/Indonesia/NIHRDI-MS636/2008 | EPI465596 \| A/Indonesia/NIHRDI-MS636/2008 | 04/01/2008 |
|  | EPI465597 \| A/Indonesia/NIHRDI-MS903/2008 | EPI465598 \| A/Indonesia/NIHRDI-MS903/2008 | 12/11/2008 |
|  | EPI465599 \| A/Indonesia/NIHRDI-MS905/2008 | EPI465600 \| A/Indonesia/NIHRDI-MS905/2008 | 12/11/2008 |
|  | EPI465601 \| A/Indonesia/NIHRDI-MS915/2008 | EPI465602 \| A/Indonesia/NIHRDI-MS915/2008 | 12/11/2008 |
|  | EPI465603 \| A/Indonesia/NIHRDI-MS917/2008 | EPI465604 \| A/Indonesia/NIHRDI-MS917/2008 | 19/11/2008 |
|  | EPI465605 \| A/Indonesia/NIHRDI-MS920/2008 | EPI465606 \| A/Indonesia/NIHRDI-MS920/2008 | 24/11/2008 |
|  | EPI465607 \| A/Indonesia/NIHRDI-MS923/2008 | EPI465608 \| A/Indonesia/NIHRDI-MS923/2008 | 05/12/2008 |
|  | EPI465670 \| A/Indonesia/NIHRDI-MS937/2009 | EPI465671 \| A/Indonesia/NIHRDI-MS937/2009 | 05/01/2009 |
|  | EPI465672 \| A/Indonesia/NIHRDI-MS938/2009 | EPI465673 \| A/Indonesia/NIHRDI-MS938/2009 | 07/01/2009 |
|  | EPI465674 \| A/Indonesia/NIHRDI-MS958/2009 | EPI465675 \| A/Indonesia/NIHRDI-MS958/2009 | 23/01/2009 |
|  | EPI465713 \| A/Indonesia/NIHRDI-MKS253/2010 | EPI465714 \| A/Indonesia/NIHRDI-MKS253/2010 | 26/04/2010 |
|  | EPI465715 \| A/Indonesia/NIHRDI-MKS222/2010 | EPI465716 \| A/Indonesia/NIHRDI-MKS222/2010 | 15/03/2010 |
|  | EPI465727 \| A/Indonesia/NIHRDI-MS249/2010 | EPI465728 \| A/Indonesia/NIHRDI-MS249/2010 | 12/04/2010 |
|  | EPI465729 \| A/Indonesia/NIHRDI-MS262/2010 | EPI465730 \| A/Indonesia/NIHRDI-MS262/2010 | 03/05/2010 |
|  | EPI465731 \| A/Indonesia/NIHRDI-MS283/2010 | EPI465732 \| A/Indonesia/NIHRDI-MS283/2010 | 31/05/2010 |
|  | EPI465733 \| A/Indonesia/NIHRDI-MS285/2010 | EPI465734 \| A/Indonesia/NIHRDI-MS285/2010 | 07/06/2010 |
| Merauke | EPI465717 \| A/Indonesia/NIHRDI-MRK195/2010 | EPI465718 \| A/Indonesia/NIHRDI-MRK195/2010 | 12/04/2010 |
|  | EPI465719 \| A/Indonesia/NIHRDI-MRK206/2010 | EPI465720 \| A/Indonesia/NIHRDI-MRK206/2010 | 26/04/2010 |
|  | EPI465721 \| A/Indonesia/NIHRDI-MRK228/2010 | EPI465722 \| A/Indonesia/NIHRDI-MRK228/2010 | 10/05/2010 |
|  | EPI465723 \| A/Indonesia/NIHRDI-MRK252/2010 | EPI465724 \| A/Indonesia/NIHRDI-MRK252/2010 | 14/06/2010 |
|  | EPI465725 \| A/Indonesia/NIHRDI-MRK255/2010 | EPI465726 \| A/Indonesia/NIHRDI-MRK255/2010 | 14/06/2010 |
