# Supplementary material for: Evolutionary study and phylodynamic pattern of human influenza A/H3N2 virus in Indonesia from 2008 to 2010
Source: PLoS One. 2018 Aug 1;13(8):e0201427. doi: 10.1371/journal.pone.0201427 (PMC6070282; doi:10.1371/journal.pone.0201427)
Supplement: S2 Table — (DOCX) [file pone.0201427.s002.docx]

| **No** | **HA Sequence** | **No** | **HA Sequence** |
| --- | --- | --- | --- |
| 1 | CY025277/A/Illinois/UR06-0567/2007 | 71 | CY062338/A/Egypt/N11352/2009 |
| 2 | CY025477/A/Illinois/UR06-0334/2007 | 72 | CY062339/A/Egypt/N11354/2009 |
| 3 | CY025851/A/Texas/UR06-0418/2007 | 73 | CY062340/A/Egypt/N11360/2009 |
| 4 | CY026019/A/Illinois/UR06-0036/2007 | 74 | CY062341/A/Egypt/N11362/2009 |
| 5 | CY026027/A/Illinois/UR06-0030/2007 | 75 | CY062342/A/Egypt/N11368/2009 |
| 6 | CY026035/A/Alabama/UR06-0482/2007 | 76 | CY062343/A/Egypt/N11380/2009 |
| 7 | CY026163/A/Colorado/UR06-0535/2007 | 77 | CY062345/A/Egypt/N11830/2009 |
| 8 | CY026259/A/Illinois/UR06-0555/2007 | 78 | CY062346/A/Egypt/N12479/2009 |
| 9 | CY026667/A/Colorado/UR06-0279/2007 | 79 | CY062347/A/Ghana/N04985/2009 |
| 10 | CY026707/A/Colorado/UR06-0454/2007 | 80 | CY062348/A/Jordan/N05371/2009 |
| 11 | CY026747/A/Illinois/UR06-0528/2007 | 81 | CY062351/A/Egypt/N13654/2009 |
| 12 | CY026771/A/Washington/UR06-0252/2007 | 82 | CY062352/A/Egypt/N13658/2009 |
| 13 | CY026787/A/California/UR06-0565/2007 | 83 | CY062353/A/Egypt/N13908/2009 |
| 14 | CY026883/A/Kentucky/UR06-0158/2007 | 84 | CY064879/A/California/VRDL147/2009 |
| 15 | CY026923/A/Ohio/UR06-0494/2007 | 85 | CY067205/A/California/VRDL291/2009 |
| 16 | CY027539/A/Colorado/UR06-022/2007 | 86 | CY067213/A/California/VRDL296/2009 |
| 17 | CY027547/A/Colorado/UR06-0024/2007 | 87 | CY067237/A/California/VRDL307/2009 |
| 18 | CY027563/A/Texas/UR06-0358/2007 | 88 | CY067245/A/California/VRDL314/2009 |
| 19 | CY027587/A/Illinois/UR06-0546/2007 | 89 | CY067253/A/California/VRDL316/2009 |
| 20 | CY027715/A/Illinois/UR06-0402/2007 | 90 | CY067921/A/California/VRDL176/2009 |
| 21 | CY028740/A/Virginia/UR06-0489/2007 | 91 | CY067953/A/California/VRDL288/2009 |
| 22 | CY032437/A/Managua/25/2007 | 92 | CY067969/A/California/VRDL339/2009 |
| 23 | CY032453/A/Managua/32/2007 | 93 | CY067977/A/California/VRDL340/2009 |
| 24 | CY032461/A/Managua/15/2007 | 94 | CY067985/A/California/VRDL342/2009 |
| 25 | CY032501/A/Managua/18/2007 | 95 | CY068033/A/California/VRDL352/2009 |
| 26 | CY034414/A/New York/UR06-0607/2007 | 96 | CY068041/A/California/VRDL354/2009 |
| 27 | CY035022/A/Brisbane/10/2007 | 97 | CY068049/A/California/VRDL355/2009 |
| 28 | CY035038/A/Pennsylvania/PIT02/2008 | 98 | CY068065/A/California/VRDL360/2009 |
| 29 | CY035086/A/Managua/3/2007 | 99 | CY068081/A/California/VRDL362/2009 |
| 30 | CY036927/A/Pennsylvania/PIT04/2008 | 100 | CY068097/A/California/VRDL368/2009 |
| 31 | CY036959/A/Ohio/UR07-0043/2008 | 101 | CY068121/A/California/VRDL375/2009 |
| 32 | CY036975/A/Ohio/UR07-0089/2008 | 102 | CY068129/A/California/VRDL396/2009 |
| 33 | CY036991/A/Kansas/UR07-0135/2008 | 103 | CY068137/A/California/VRDL159/2009 |
| 34 | CY037479/A/Ohio/UR07-0005/2008 | 104 | CY068217/A/California/VRDL173/2009 |
| 35 | CY037519/A/Ohio/UR07-0031/2008 | 105 | CY068289/A/California/VRDL247/2009 |
| 36 | CY037551/A/Ohio/UR07-0058/2008 | 106 | CY068353/A/California/VRDL263/2009 |
| 37 | CY037591/A/Kentucky/UR07-0108/2008 | 107 | CY068409/A/California/VRDL277/2009 |
| 38 | CY037599/A/Kansas/UR07-0110/2008 | 108 | CY068433/A/California/VRDL285/2009 |
| 39 | CY037727/A/Florida/UR07-0102/2008 | 109 | CY068457/A/California/VRDL305/2009 |
| 40 | CY037855/A/Ohio/UR07-0023/2008 | 110 | CY068465/A/California/VRDL308/2009 |
| 41 | CY037887/A/Ohio/UR07-0126/2008 | 111 | CY068545/A/California/VRDL322/2009 |
| 42 | CY038519/A/Managua/112.01/2007 | 112 | CY068577/A/California/VRDL331/2009 |
| 43 | CY040098/A/Taiwan/70120/2008 | 113 | CY068585/A/California/VRDL332/2009 |
| 44 | CY040122/A/Taiwan/72106/2007 | 114 | CY068609/A/California/VRDL335/2009 |
| 45 | CY041474/A/Florida/UR07-0154/2008 | 115 | CY068625/A/California/VRDL338/2009 |
| 46 | CY044492/A/Boston/14/2007 | 116 | CY068758/A/California/VRDL203/2009 |
| 47 | CY044508/A/Boston/20/2008 | 117 | CY068806/A/California/VRDL246/2009 |
| 48 | CY044516/A/Boston/21/2008 | 118 | CY068822/A/California/VRDL381/2009 |
| 49 | CY044572/A/Boston/36/2008 | 119 | CY068830/A/California/VRDL383/2009 |
| 50 | CY044604/A/Boston/42/2008 | 120 | CY068838/A/California/VRDL384/2009 |
| 51 | CY044804/A/Boston/79/2008 | 121 | CY068854/A/California/VRDL390/2009 |
| 52 | CY044836/A/Boston/94/2008 | 122 | CY069421/A/New Mexico/WRAIR1139P/2009 |
| 53 | CY044844/A/Boston/96/2008 | 123 | CY070927/A/Managua/5806.01/2010 |
| 54 | CY044852/A/Boston/97/2008 | 124 | CY073757/A/California/VRDL363/2009 |
| 55 | CY050564/A/New York/3104/2009 | 125 | CY074683/A/Managua/5906.01/2010 |
| 56 | CY050700/A/New York/3279/2009 | 126 | CY074803/A/Managua/3715.02/2010 |
| 57 | CY050708/A/New York/3280/2009 | 127 | CY074827/A/Managua/3433.02/2010 |
| 58 | CY050820/A/New York/3738/2009 | 128 | CY074843/A/Managua/1166.03/2010 |
| 59 | CY053652/A/Novosibirsk/1211/2009 | 129 | CY074851/A/Managua/5543.01/2010 |
| 60 | CY053666/A/Novosibirsk/628/2009 | 130 | CY074867/A/Managua/4134.04/2010 |
| 61 | CY055083/A/New York/3687/2009 | 131 | CY074942/A/Thailand/CU-H1071/2009 |
| 62 | CY061898/A/Australia/19/2009 | 132 | CY074950/A/Thailand/CU-H1285/2010 |
| 63 | CY062329/A/Djibouti/N04765/2009 | 133 | CY074958/A/Thailand/CU-H1443/2010 |
| 64 | CY062330/A/Djibouti/N04767/2009 | 134 | CY074966/A/Thailand/CU-H1817/2010 |
| 65 | CY062331/A/Djibouti/N04775/2009 | 135 | CY080450/A/New York/3745/2009 |
| 66 | CY062332/A/Djibouti/N04776/2009 | 136 | CY080475/A/New York/3519/2009 |
| 67 | CY062334/A/Egypt/N04877/2009 | 137 | CY080491/A/New York/3932/2009 |
| 68 | CY062335/A/Egypt/N08906/2009 | 138 | CY080531/A/Australia/46/2009 |
| 69 | CY062336/A/Egypt/N08920/2009 | 139 | CY080547/A/Australia/30/2009 |
| 70 | CY062337/A/Egypt/N11008/2009 | 140 | CY080563/A/Australia/55/2009 |

| **No** | **HA Sequence** | **No** | **HA Sequence** |
| --- | --- | --- | --- |
| 141 | CY081428/A/Perth/16/2009 | 211 | CY118131/A/Malaysia/1959476/2008 |
| 142 | CY084334/A/New York/6630/2009 | 212 | CY118151/A/Malaysia/1997829/2008 |
| 143 | CY087705/A/Uganda/MUWRP-062/2009 | 213 | CY118159/A/Malaysia/1997831/2008 |
| 144 | CY087712/A/Uganda/MUWRP-065/2009 | 214 | CY118946/A/Malaysia/1755590/2007 |
| 145 | CY087718/A/Uganda/MUWRP-070/2009 | 215 | CY118954/A/Malaysia/1756502/2007 |
| 146 | CY087724/A/Uganda/MUWRP-074/2009 | 216 | CY118962/A/Malaysia/1756937/2007 |
| 147 | CY088774/A/Managua/511.03/2010 | 217 | CY118970/A/Malaysia/1767091/2007 |
| 148 | CY088843/A/Managua/3345.02/2010 | 218 | CY118986/A/Malaysia/1773914/2007 |
| 149 | CY088883/A/Managua/4355.03/2010 | 219 | CY118994/A/Malaysia/1779036/2007 |
| 150 | CY088907/A/Managua/3039.01/2010 | 220 | CY119002/A/Malaysia/1781328/2007 |
| 151 | CY088923/A/Managua/5801.02/2010 | 221 | CY119034/A/Malaysia/1963888/2008 |
| 152 | CY088931/A/Managua/4747.04/2010 | 222 | CY119042/A/Malaysia/1965896/2008 |
| 153 | CY088939/A/Managua/38.01/2010 | 223 | CY119050/A/Malaysia/1770921/2007 |
| 154 | CY088955/A/Managua/3622.02/2010 | 224 | CY120890/A/Colorado/3005/2012 |
| 155 | CY088963/A/Managua/5898.02/2010 | 225 | CY121077/A/Victoria/210/2009 |
| 156 | CY088979/A/Managua/3408.02/2010 | 226 | CY121632/A/Uruguay/716/2007 |
| 157 | CY088987/A/Managua/3659.02/2010 | 227 | CY121792/A/Brisbane/11/2010 |
| 158 | CY088995/A/Managua/1685.01/2010 | 228 | EU100721/2007726992A/North Carolina/01/2007 |
| 159 | CY089011/A/Managua/4604.04/2010 | 229 | EU100722/2007726993A/North Carolina/02/2007 |
| 160 | CY089019/A/Managua/5870.02/2010 | 230 | EU100723/2007726998A/Virginia/08/2006 |
| 161 | CY089027/A/Managua/4855.03/2010 | 231 | EU199250/A/Brisbane/10/2007 |
| 162 | CY089733/A/Boston/40/2009 | 232 | EU199256/2007727078A/Colorado/06/2006 |
| 163 | CY089765/A/Boston/97/2009 | 233 | EU199275/2007727477A/Idaho/03/2007 |
| 164 | CY090877/A/Sydney/DD2-02/2010 | 234 | EU199277/2007727657A/New Jersey/03/2007 |
| 165 | CY091831/A/Guangdong/17/2007 | 235 | EU199280/2007727691A/Memphis/07/2007 |
| 166 | CY092241/A/California/NHRC0001/2007 | 236 | EU199281/2007727907A/Missouri/03/2007 |
| 167 | CY092297/A/Managua/58-11/2011 | 237 | EU199284/2007728934A/Virginia/01/2007 |
| 168 | CY092361/A/California/VRDL242/2009 | 238 | EU199285/2007728936A/Virginia/02/2007 |
| 169 | CY092369/A/California/VRDL255/2009 | 239 | EU199346/2007728527A/Wisconsin/26/2007 |
| 170 | CY093248/A/Texas/WRAIR1239P/2009 | 240 | EU199347/2007729543A/Texas/10/2007 |
| 171 | CY093335/A/Guam/WRAIR1557P/2009 | 241 | EU199358/2007730274A/Minnesota/18/2007 |
| 172 | CY093351/A/Kuwait/WRAIR1561P/2009 | 242 | EU199360/2007728400A/South Carolina/01/2007 |
| 173 | CY093375/A/Mexico City/WRAIR1752T/2010 | 243 | EU199365/2007730265A/Vermont/05/2007 |
| 174 | CY093391/A/Belgrade/WRAIR2379N/2010 | 244 | EU199377/2007728333A/South Dakota/01/2007 |
| 175 | CY093399/A/Belgrade/WRAIR2956T/2010 | 245 | EU199378/2007728903A/Minnesota/17/2007 |
| 176 | CY100073/A/Singapore/GP2240/2009 | 246 | EU516030/2007731807A/Georgia/05/2007 |
| 177 | CY100075/A/Singapore/GP3303/2009 | 247 | EU516064/2008704020A/Texas/63/2007 |
| 178 | CY100083/A/Singapore/GP586/2009 | 248 | EU516068/2008703985A/Kentucky/01/2007 |
| 179 | CY100085/A/Singapore/GP638/2009 | 249 | EU516069/2008703935A/Colorado/22/2007 |
| 180 | CY100091/A/Singapore/GP3268/2010 | 250 | EU516216/2008704066A/Montana/08/2007 |
| 181 | CY100099/A/Singapore/GP3727/2010 | 251 | EU516217/2008704325A/North Carolina/05/2007 |
| 182 | CY104444/A/HaNoi/Q118/2007 | 252 | EU516219/2008704459A/Virginia/04/2007 |
| 183 | CY104524/A/HaNoi/TX320/2007 | 253 | EU659832/A/British Columbia/0613/2007 |
| 184 | CY104540/A/HaNoi/TX327/2007 | 254 | EU659844/A/British Columbia/0229/2007 |
| 185 | CY105478/A/HaNoi/BM171/2007 | 255 | EU659845/A/British Columbia/0222/2007 |
| 186 | CY105502/A/HaNoi/BT251/2007 | 256 | EU716426/2007731384A/Uruguay/716/2007 |
| 187 | CY105710/A/HaNoi/Q499/2007 | 257 | EU716428/2007731384A/Uruguay/716/2007 |
| 188 | CY105822/A/DaNang/DN453/2008 | 258 | EU716429/2007731365A/Uruguay/716/2007 |
| 189 | CY106576/A/Hong Kong/H090-662-V10/2009 | 259 | EU716453/2008740290A/Wisconsin/08/2008 |
| 190 | CY106680/A/Hong Kong/H090-707-V10/2009 | 260 | EU716470/2008707609A/Wisconsin/06/2008 |
| 191 | CY106928/A/Hong Kong/H090-756-V10/2009 | 261 | EU716502/2008707598A/Wisconsin/02/2008 |
| 192 | CY110774/A/Vladivostok/59/2012 | 262 | EU779512/2008707289A/Memphis/30/2008 |
| 193 | CY110775/A/Vladivostok/81/2012 | 263 | EU779522/2008708313A/Virginia/02/2008 |
| 194 | CY110776/A/Vladivostok/99/2012 | 264 | EU779530/2008740857A/Missouri/01/2008 |
| 195 | CY111009/A/Ontario/007/2010 | 265 | EU852001/2008740551A/North Carolina/01/2008 |
| 196 | CY111035/A/Quebec/028/2011 | 266 | EU885503/A/Memphis/28/2008 |
| 197 | CY111038/A/Quebec/032/2011 | 267 | EU885509/A/New York/07/2008 |
| 198 | CY113037/A/Netherlands/761/2009 | 268 | EU885514/A/Washington/03/2008 |
| 199 | CY114413/A/Netherlands/348/2007 | 269 | EU885522/A/Washington/04/2008 |
| 200 | CY114563/A/Saint-Petersburg/RII01/2012 | 270 | EU885528/A/Illinois/09/2008 |
| 201 | CY115464/A/Hong Kong/H090-662-V10/2009 | 271 | EU885534/A/Minnesota/06/2008 |
| 202 | CY115504/A/Hong Kong/H090-688-V10/2009 | 272 | EU914857/A/Ohio/11/2008 |
| 203 | CY115560/A/Hong Kong/H090-707-V10/2009 | 273 | FJ179352/A/Minnesota/16/2008 |
| 204 | CY115768/A/Hong Kong/H090-752-V10/2009 | 274 | FJ179354/A/Minnesota/14/2008 |
| 205 | CY115784/A/Hong Kong/H090-756-V10/2009 | 275 | FJ686930/A/Missouri/05/2008 |
| 206 | CY118051/A/Malaysia/1768409/2007 | 276 | FJ769863/A/Tehran/631/2007 |
| 207 | CY118059/A/Malaysia/1769312/2007 | 277 | FJ769864/A/Kermanshah/687/2007 |
| 208 | CY118067/A/Malaysia/1777545/2007 | 278 | FJ769915/A/Tehran/379/2007 |
| 209 | CY118075/A/Malaysia/1779027/2007 | 279 | FJ912992/A/Thailand/CU379/2008 |
| 210 | CY118123/A/Malaysia/1918618/2007 | 280 | FJ966245/A/Victoria/502/2009 |

| **No** | **HA Sequence** |
| --- | --- |
| 282 | GQ293083/A/Philippines/16/2009 |
| 283 | GQ293085/A/Philippines/5/2009 |
| 284 | GQ293087/A/Singapore/39/2009 |
| 285 | GQ385818/A/Hawaii/02/2009 |
| 286 | GQ385862/A/Texas/03/2009 |
| 287 | GQ385882/A/Hawaii/14/2009 |
| 288 | GQ385891/A/Pennsylvania/02/2009 |
| 289 | GQ385897/A/Hawaii/06/2009 |
| 290 | GQ385912/A/Hawaii/15/2009 |
| 291 | GQ894995/A/North Carolina/14/2009 |
| 292 | GQ895022/A/Texas/40/2009 |
| 293 | GQ895044/A/Kansas/04/2009 |
| 294 | GQ895050/A/South Carolina/14/2009 |
| 295 | GQ902793/A/Thailand/CU-B4/2009 |
| 296 | GQ902817/A/Thailand/CU-B590/2009 |
| 297 | GQ902825/A/Thailand/CU-B657/2009 |
| 298 | GQ983548/A/Thailand/CU-B106/2009 |
| 299 | GU271974/A/Thailand/CU-B1672/2009 |
| 300 | GU931683/Singapore/DSO20090004/2009 |
| 301 | GU931684/Singapore/DSO20090008/2009 |
| 302 | HQ315822/A/Stockholm/4/2010 |
| 303 | HQ703347/A/Niigata/1143/2010 |
| 304 | HQ703348/A/Niigata/1144/2010 |
| 305 | HQ703349/A/Niigata/1146/2010 |
| 306 | HQ703350/A/Niigata/1147/2010 |
| 307 | HQ703351/A/Niigata/1148/2010 |
| 308 | HQ703352/A/Niigata/1149/2010 |
| 309 | HQ703353/A/Niigata/1150/2010 |
| 310 | HQ853209/A/Peru/CIS1578/2010 |
| 311 | JN940429/A/Novosibirsk/1832/2011 |
| 312 | JN940431/A/Novosibirsk/1927/2011 |
| 313 | JQ396182/A/Kenya/80/2010 |
| 314 | JQ396183/A/Kenya/091/2010 |
| 315 | JQ396185/A/Kenya/096/2010 |
| 316 | JQ655462/A/Nizhniy Novgorod/668/2008 |
| 317 | JQ655463/A/Astrahan/10/2007 |
| 318 | JQ988027/A/Penza/55/2008 |
| 319 | JQ988039/A/Moscow/21/2009 |
| 320 | JQ988045/A/Vladivostok/10/2011 |

| **No** | **NA Sequence** | **No** | **NA Sequence** |
| --- | --- | --- | --- |
| 1 | CY024910/A/USA/AF1086/2007 | 71 | CY040068/A/Taiwan/2361/2007 |
| 2 | CY025415/A/Vermont/UR06-0524/2007 | 72 | CY040124/A/Taiwan/72106/2007 |
| 3 | CY025423/A/California/UR06-0347/2007 | 73 | CY040220/A/Managua/3012.01/2007 |
| 4 | CY025479/A/Illinois/UR06-0334/2007 | 74 | CY041468/A/Florida/UR07-0095/2008 |
| 5 | CY025503/A/Vermont/UR06-0484/2007 | 75 | CY044383/A/New York/UR07-0160/2008 |
| 6 | CY025542/A/Vermont/UR06-0448/2007 | 76 | CY044471/A/Boston/9/2008 |
| 7 | CY025709/A/Illinois/UR06-0478/2007 | 77 | CY044478/A/Boston/10/2008 |
| 8 | CY025733/A/New York/UR06-0529/2007 | 78 | CY044606/A/Boston/42/2008 |
| 9 | CY025869/A/Vermont/UR06-0483/2007 | 79 | CY044654/A/Boston/48/2008 |
| 10 | CY025877/A/Vermont/UR06-0470/2007 | 80 | CY044694/A/Boston/55/2008 |
| 11 | CY025885/A/Vermont/UR06-0486/2007 | 81 | CY044774/A/Boston/66/2008 |
| 12 | CY025901/A/Illinois/UR06-0436/2007 | 82 | CY044814/A/Boston/81/2008 |
| 13 | CY026037/A/Alabama/UR06-0482/2007 | 83 | CY044838/A/Boston/94/2008 |
| 14 | CY026165/A/Colorado/UR06-0535/2007 | 84 | CY044846/A/Boston/96/2008 |
| 15 | CY026253/A/Colorado/UR06-0558/2007 | 85 | CY044854/A/Boston/97/2008 |
| 16 | CY026261/A/Illinois/UR06-0555/2007 | 86 | CY050139/A/Qingdao/1046/2009 |
| 17 | CY026309/A/Virginia/UR06-0021/2007 | 87 | CY050454/A/New York/1670/2009 |
| 18 | CY026669/A/Colorado/UR06-0279/2007 | 88 | CY050462/A/New York/1671/2009 |
| 19 | CY026749/A/Illinois/UR06-0528/2007 | 89 | CY050558/A/New York/3103/2009 |
| 20 | CY026789/A/California/UR06-0565/2007 | 90 | CY050566/A/New York/3104/2009 |
| 21 | CY026829/A/Oregon/UR06-0450/2007 | 91 | CY050702/A/New York/3279/2009 |
| 22 | CY026845/A/Oregon/UR06-0289/2007 | 92 | CY050710/A/New York/3280/2009 |
| 23 | CY026885/A/Kentucky/UR06-0158/2007 | 93 | CY055085/A/New York/3687/2009 |
| 24 | CY027109/A/Oregon/UR06-0389/2007 | 94 | CY058758/A/New York/3316/2009 |
| 25 | CY027197/A/Vermont/UR06-0469/2007 | 95 | CY061892/A/Australia/2/2009 |
| 26 | CY027541/A/Colorado/UR06-022/2007 | 96 | CY061900/A/Australia/19/2009 |
| 27 | CY027549/A/Colorado/UR06-0024/2007 | 97 | CY064865/A/California/VRDL145/2009 |
| 28 | CY027589/A/Illinois/UR06-0546/2007 | 98 | CY066513/A/Australia/18/2009 |
| 29 | CY027717/A/Illinois/UR06-0402/2007 | 99 | CY067215/A/California/VRDL296/2009 |
| 30 | CY027869/A/Virginia/UR06-0580/2007 | 100 | CY067971/A/California/VRDL339/2009 |
| 31 | CY028301/A/Ohio/UR06-0256/2007 | 101 | CY067979/A/California/VRDL340/2009 |
| 32 | CY028373/A/Oregon/UR06-0221/2007 | 102 | CY068011/A/California/VRDL345/2009 |
| 33 | CY028477/A/Texas/UR06-0603/2007 | 103 | CY068019/A/California/VRDL349/2009 |
| 34 | CY031557/A/Kentucky/UR06-0571/2007 | 104 | CY068027/A/California/VRDL351/2009 |
| 35 | CY032471/A/Managua/16/2007 | 105 | CY068035/A/California/VRDL352/2009 |
| 36 | CY033147/A/Managua/30/2007 | 106 | CY068051/A/California/VRDL355/2009 |
| 37 | CY033459/A/Vermont/UR06-0329/2007 | 107 | CY068083/A/California/VRDL362/2009 |
| 38 | CY034408/A/Vermont/UR06-0512/2007 | 108 | CY068099/A/California/VRDL368/2009 |
| 39 | CY034416/A/New York/UR06-0607/2007 | 109 | CY068107/A/California/VRDL373/2009 |
| 40 | CY035096/A/Managua/26/2007 | 110 | CY068123/A/California/VRDL375/2009 |
| 41 | CY035184/A/Pennsylvania/PIT31/2008 | 111 | CY068139/A/California/VRDL159/2009 |
| 42 | CY036961/A/Ohio/UR07-0043/2008 | 112 | CY068171/A/California/VRDL165/2009 |
| 43 | CY036985/A/Florida/UR07-0101/2008 | 113 | CY068219/A/California/VRDL173/2009 |
| 44 | CY036993/A/Kansas/UR07-0135/2008 | 114 | CY068299/A/California/VRDL248/2009 |
| 45 | CY037489/A/Kansas/UR07-0007/2008 | 115 | CY068331/A/California/VRDL260/2009 |
| 46 | CY037505/A/California/UR07-0019/2008 | 116 | CY068355/A/California/VRDL263/2009 |
| 47 | CY037529/A/Kentucky/UR07-0037/2008 | 117 | CY068371/A/California/VRDL266/2009 |
| 48 | CY037537/A/Kentucky/UR07-0041/2008 | 118 | CY068395/A/California/VRDL272/2009 |
| 49 | CY037561/A/Kentucky/UR07-0068/2008 | 119 | CY068451/A/California/VRDL294/2009 |
| 50 | CY037569/A/Kentucky/UR07-0081/2008 | 120 | CY068499/A/California/VRDL313/2009 |
| 51 | CY037585/A/Kentucky/UR07-0107/2008 | 121 | CY068507/A/California/VRDL315/2009 |
| 52 | CY037601/A/Kansas/UR07-0110/2008 | 122 | CY068515/A/California/VRDL317/2009 |
| 53 | CY037609/A/New York/UR07-0111/2008 | 123 | CY068531/A/California/VRDL319/2009 |
| 54 | CY037625/A/Florida/UR07-0146/2008 | 124 | CY068571/A/California/VRDL329/2009 |
| 55 | CY037633/A/New York/UR07-0153/2008 | 125 | CY068579/A/California/VRDL331/2009 |
| 56 | CY037705/A/Kentucky/UR07-0072/2008 | 126 | CY068603/A/California/VRDL334/2009 |
| 57 | CY037745/A/New York/UR07-0133/2008 | 127 | CY068627/A/California/VRDL338/2009 |
| 58 | CY037793/A/Kentucky/UR07-0028/2008 | 128 | CY068688/A/California/VRDL181/2009 |
| 59 | CY037809/A/Kansas/UR07-0047/2008 | 129 | CY068704/A/California/VRDL184/2009 |
| 60 | CY037833/A/Kentucky/UR07-0124/2008 | 130 | CY068744/A/California/VRDL199/2009 |
| 61 | CY037841/A/Kansas/UR07-0129/2008 | 131 | CY068808/A/California/VRDL246/2009 |
| 62 | CY037873/A/Kentucky/UR07-0082/2008 | 132 | CY068840/A/California/VRDL384/2009 |
| 63 | CY038561/A/Managua/1867.01/2007 | 133 | CY068856/A/California/VRDL390/2009 |
| 64 | CY038785/A/Mississippi/UR07-0003/2008 | 134 | CY068872/A/California/VRDL392/2009 |
| 65 | CY038825/A/Kentucky/UR07-0069/2008 | 135 | CY069423/A/New Mexico/WRAIR1139P/2009 |
| 66 | CY038865/A/Kentucky/UR07-0149/2008 | 136 | CY070969/A/New York/20342/2010 |
| 67 | CY038873/A/Florida/UR07-0150/2008 | 137 | CY072192/A/California/VRDL347/2009 |
| 68 | CY039105/A/Mississippi/UR07-0042/2008 | 138 | CY072216/A/New York/20343/2010 |
| 69 | CY039433/A/Kentucky/UR07-0116/2008 | 139 | CY073759/A/California/VRDL363/2009 |
| 70 | CY039441/A/Managua/4348.01/2007 | 140 | CY074901/A/Managua/4203.02/2010 |

| **No** | **NA Sequence** | **No** | **NA Sequence** |
| --- | --- | --- | --- |
| 141 | CY074952/A/Thailand/CU-H1285/2010 | 211 | CY100094/A/Singapore/GP3269/2010 |
| 142 | CY074960/A/Thailand/CU-H1443/2010 | 212 | CY100100/A/Singapore/GP3727/2010 |
| 143 | CY077427/A/California/VRDL356/2009 | 213 | CY100104/A/Singapore/GP3961/2010 |
| 144 | CY080452/A/New York/3745/2009 | 214 | CY100106/A/Singapore/GP4111/2010 |
| 145 | CY080461/A/New York/3421/2009 | 215 | CY100110/A/Singapore/GP4278/2010 |
| 146 | CY080469/A/New York/3470/2009 | 216 | CY100112/A/Singapore/GP4397/2010 |
| 147 | CY080477/A/New York/3519/2009 | 217 | CY100114/A/Singapore/GP4453/2010 |
| 148 | CY080533/A/Australia/46/2009 | 218 | CY100118/A/Singapore/GP4555/2010 |
| 149 | CY080541/A/Australia/22/2009 | 219 | CY100122/A/Singapore/TT381/2010 |
| 150 | CY080549/A/Australia/30/2009 | 220 | CY100124/A/Singapore/TT382/2010 |
| 151 | CY080557/A/Australia/34/2009 | 221 | CY100126/A/Singapore/TT383/2010 |
| 152 | CY081432/A/Cambodia/NHRCC00003/2009 | 222 | CY104446/A/HaNoi/Q118/2007 |
| 153 | CY081440/A/Cambodia/NHRCC00007/2009 | 223 | CY104494/A/HaNoi/Q672/2007 |
| 154 | CY081448/A/Cambodia/NHRCC00008/2009 | 224 | CY104542/A/HaNoi/TX327/2007 |
| 155 | CY081464/A/Cambodia/NHRCC00001/2009 | 225 | CY105512/A/HaNoi/GS0737/2007 |
| 156 | CY081472/A/Cambodia/NHRCC00002/2009 | 226 | CY105696/A/HaNoi/Q464/2007 |
| 157 | CY081480/A/Cambodia/NHRCC00006/2009 | 227 | CY105704/A/HaNoi/Q492/2007 |
| 158 | CY084336/A/New York/6630/2009 | 228 | CY105712/A/HaNoi/Q499/2007 |
| 159 | CY087320/A/Uganda/MUWRP-002/2008 | 229 | CY105736/A/HaNoi/Q638/2007 |
| 160 | CY087352/A/Uganda/MUWRP-007/2008 | 230 | CY105760/A/TayNguyen/TN341/2007 |
| 161 | CY087383/A/Uganda/MUWRP-011/2008 | 231 | CY105768/A/TayNguyen/TN367/2007 |
| 162 | CY087422/A/Uganda/MUWRP-016/2008 | 232 | CY106578/A/Hong Kong/H090-662-V10/2009 |
| 163 | CY087592/A/Uganda/MUWRP-040/2008 | 233 | CY106586/A/Hong Kong/H090-669-V10/2009 |
| 164 | CY087608/A/Uganda/MUWRP-042/2008 | 234 | CY106594/A/Hong Kong/H090-671-V10/2009 |
| 165 | CY087670/A/Uganda/MUWRP-050/2008 | 235 | CY106682/A/Hong Kong/H090-707-V10/2009 |
| 166 | CY087692/A/Uganda/MUWRP-058/2009 | 236 | CY106706/A/Hong Kong/H090-720-V10/2009 |
| 167 | CY087699/A/Uganda/MUWRP-061/2009 | 237 | CY106714/A/Hong Kong/H090-720-V20/2009 |
| 168 | CY087707/A/Uganda/MUWRP-062/2009 | 238 | CY106722/A/Hong Kong/H090-720-V21/2009 |
| 169 | CY087714/A/Uganda/MUWRP-065/2009 | 239 | CY106730/A/Hong Kong/H090-720-V31/2009 |
| 170 | CY087720/A/Uganda/MUWRP-070/2009 | 240 | CY106930/A/Hong Kong/H090-756-V10/2009 |
| 171 | CY087726/A/Uganda/MUWRP-074/2009 | 241 | CY106938/A/Hong Kong/H090-763-V10/2009 |
| 172 | CY087734/A/Uganda/MUWRP-075/2009 | 242 | CY106946/A/Hong Kong/H090-763-V23/2009 |
| 173 | CY087742/A/Uganda/MUWRP-079/2009 | 243 | CY106986/A/Hong Kong/H090-781-V10/2009 |
| 174 | CY087749/A/Uganda/MUWRP-086/2009 | 244 | CY111136/A/Boston/DOA02/2011 |
| 175 | CY088853/A/Managua/2654.01/2010 | 245 | CY111144/A/Boston/DOA03/2011 |
| 176 | CY088885/A/Managua/4355.03/2010 | 246 | CY111152/A/Boston/DOA04/2011 |
| 177 | CY088893/A/Managua/1260.02/2010 | 247 | CY111176/A/Boston/DOA07/2011 |
| 178 | CY088941/A/Managua/38.01/2010 | 248 | CY111224/A/Boston/DOA10/2011 |
| 179 | CY088957/A/Managua/3622.02/2010 | 249 | CY111248/A/Boston/DOA20/2011 |
| 180 | CY089021/A/Managua/5870.02/2010 | 250 | CY111304/A/Boston/DOA48/2011 |
| 181 | CY089631/A/New York/3750/2009 | 251 | CY111320/A/Boston/DOA52/2011 |
| 182 | CY089767/A/Boston/97/2009 | 252 | CY111360/A/Boston/DOA67/2011 |
| 183 | CY089775/A/Boston/99/2009 | 253 | CY111448/A/Boston/DOA82/2011 |
| 184 | CY090871/A/Sydney/DD2-01/2010 | 254 | CY113039/A/Netherlands/761/2009 |
| 185 | CY090879/A/Sydney/DD2-02/2010 | 255 | CY114511/A/Netherlands/034/2010 |
| 186 | CY091559/A/Texas/NHRC0001/2011 | 256 | CY114535/A/Petrozavodsk/RII01/2012 |
| 187 | CY091575/A/Illinois/NHRC0002/2011 | 257 | CY114540/A/Novosibirsk/RII08/2012 |
| 188 | CY091583/A/California/NHRC0001/2011 | 258 | CY114555/A/Astrakhan/RII65/2011 |
| 189 | CY092283/A/California/NHRC0002/2011 | 259 | CY114560/A/Novosibirsk/RII09/2012 |
| 190 | CY092307/A/Managua/2492.04/2010 | 260 | CY114565/A/Saint-Petersburg/RII01/2012 |
| 191 | CY093119/A/Managua/5744.03/2010 | 261 | CY115466/A/Hong Kong/H090-662-V10/2009 |
| 192 | CY093218/A/Dakar/WRAIR0013N/2009 | 262 | CY115474/A/Hong Kong/H090-669-V10/2009 |
| 193 | CY093226/A/District of Columbia/WRAIR0300/2010 | 263 | CY115482/A/Hong Kong/H090-671-V10/2009 |
| 194 | CY093249/A/Texas/WRAIR1239P/2009 | 264 | CY115562/A/Hong Kong/H090-707-V10/2009 |
| 195 | CY093321/A/Kyrgyzstan/WRAIR1256P/2008 | 265 | CY115586/A/Hong Kong/H090-720-V10/2009 |
| 196 | CY093353/A/Kuwait/WRAIR1561P/2009 | 266 | CY115594/A/Hong Kong/H090-720-V20/2009 |
| 197 | CY093385/A/District of Columbia/WRAIR1753P/2010 | 267 | CY115602/A/Hong Kong/H090-720-V21/2009 |
| 198 | CY093489/A/Mexico City/WRAIR3570T/2010 | 268 | CY115610/A/Hong Kong/H090-720-V31/2009 |
| 199 | CY093497/A/Mexico City/WRAIR3570T/2010 | 269 | CY115786/A/Hong Kong/H090-756-V10/2009 |
| 200 | CY093561/A/Mexico City/WRAIR4139N/2010 | 270 | CY115794/A/Hong Kong/H090-763-V10/2009 |
| 201 | CY098067/A/Managua/5828.02/2010 | 271 | CY115802/A/Hong Kong/H090-763-V23/2009 |
| 202 | CY098075/A/Managua/3587.02/2010 | 272 | CY116637/A/Tbilisi/GNCDC0485/2012 |
| 203 | CY098083/A/Managua/1645.03/2010 | 273 | CY116639/A/Tbilisi/GNCDC0557/2012 |
| 204 | CY100074/A/Singapore/GP2240/2009 | 274 | CY116641/A/Tbilisi/GNCDC0577/2012 |
| 205 | CY100076/A/Singapore/GP3303/2009 | 275 | CY116701/A/Boston/DOA16/2011 |
| 206 | CY100084/A/Singapore/GP586/2009 | 276 | CY118948/A/Malaysia/1755590/2007 |
| 207 | CY100086/A/Singapore/GP638/2009 | 277 | CY118956/A/Malaysia/1756502/2007 |
| 208 | CY100088/A/Singapore/N1604/2009 | 278 | CY118964/A/Malaysia/1756937/2007 |
| 209 | CY100090/A/Singapore/N1607/2009 | 279 | CY118972/A/Malaysia/1767091/2007 |
| 210 | CY100092/A/Singapore/GP3268/2010 | 280 | CY118996/A/Malaysia/1779036/2007 |

| **No** | **NA Sequence** | **No** | **NA Sequence** |
| --- | --- | --- | --- |
| 281 | CY119036/A/Malaysia/1963888/2008 | 348 | GQ385821/A/Minnesota/37/2008 |
| 282 | CY119044/A/Malaysia/1965896/2008 | 349 | GQ385823/A/New Hampshire/04/2009 |
| 283 | CY119052/A/Malaysia/1770921/2007 | 350 | GQ385836/A/Colorado/05/2009 |
| 284 | CY121079/A/Victoria/210/2009 | 351 | GQ385848/A/Wisconsin/03/2009 |
| 285 | CY121498/A/Perth/10/2010 | 352 | GQ385861/A/Texas/03/2009 |
| 286 | CY121754/A/Finland/97/2009 | 353 | GQ385863/A/Colorado/01/2009 |
| 287 | CY121778/A/Finland/97/2009 | 354 | GQ385873/A/South Carolina/07/2008 |
| 288 | CY121794/A/Brisbane/11/2010 | 355 | GQ385903/A/Virginia/02/2009 |
| 289 | CY121802/A/Rhode Island/01/2010 | 356 | GQ410126/A/Taiwan/F1963/2009 |
| 290 | EU100648/2007726993A/North Carolina/02/2007 | 357 | GQ410127/A/Taiwan/F1965/2009 |
| 291 | EU100650/2007726896A/Washington/1/2007 | 358 | GQ410128/A/Taiwan/F1967/2009 |
| 292 | EU100651/2007726569A/Washington/10/2006 | 359 | GQ410130/A/Taiwan/F298/2009 |
| 293 | EU100652/2007726112A/Washington/11/2006 | 360 | GQ410137/A/Taiwan/2428/2008 |
| 294 | EU100653/2007726200A/Wisconsin/42/2006 | 361 | GQ410138/A/Taiwan/2557/2009 |
| 295 | EU199417/2007730265A/Vermont/05/2007 | 362 | GQ895013/A/Colorado/15/2008 |
| 296 | EU199418/2007728333A/South Dakota/01/2007 | 363 | GQ895023/A/Texas/40/2009 |
| 297 | EU199419/2007729886A/Idaho/09/2007 | 364 | GQ895045/A/Kansas/04/2009 |
| 298 | EU199425/2007727509A/Colorado/14/2007 | 365 | GQ895051/A/South Carolina/14/2009 |
| 299 | EU199433/2007729777A/Rhode Island/03/2007 | 366 | GQ902795/A/Thailand/CU-B4/2009 |
| 300 | EU516039/2007730496A/Michigan/07/2007 | 367 | GQ983550/A/Thailand/CU-B106/2009 |
| 301 | EU516104/2007726147A/MARYLAND/10/2006 | 368 | GU271976/A/Thailand/CU-B1672/2009 |
| 302 | EU516167/2008703985A/Kentucky/01/2007 | 369 | GU271984/A/Thailand/CU-B1697/2009 |
| 303 | EU516169/2007728158A/Pennsylvania/4/2007 | 370 | GU907119/A/Nanjing/1/2009 |
| 304 | EU516178/2007732434A/Texas/33/2007 | 371 | HQ214370/A/Isiolo/7513/2008 |
| 305 | EU516187/2007727560A/Washington/14/2007 | 372 | HQ214372/A/Kericho/7533/2008 |
| 306 | EU516192/2007728898A/Alaska/06/2007 | 373 | HQ214373/A/Kericho/7534/2008 |
| 307 | EU516195/2008704066A/Montana/08/2007 | 374 | HQ214374/A/Kisii/7537/2008 |
| 308 | EU516225/2008704450A/Wisconsin/32/2007 | 375 | HQ214376/A/Kisii/7546/2008 |
| 309 | EU516229/2008704775A/California/35/2007 | 376 | HQ214377/A/Kisii/7548/2008 |
| 310 | EU516231/2008704953A/Tennessee/11/2007 | 377 | HQ214379/A/Kisii/7552/2008 |
| 311 | EU516233/2008704824A/Arizona/12/2007 | 378 | HQ214381/A/Kisii/7555/2008 |
| 312 | EU516235/2008704325A/North Carolina/05/2007 | 379 | HQ214382/A/Kisii/7556/2008 |
| 313 | EU566971/2008705288A/Arkansas/02/2008 | 380 | HQ214385/A/Kisii/7566/2008 |
| 314 | EU566985/2008704974A/New Jersey/21/2007 | 381 | HQ214390/A/Mbagathi/7593/2008 |
| 315 | EU566997/2008705290A/Arkansas/04/2008 | 382 | HQ214391/A/Kisumu/7597/2008 |
| 316 | EU625366/A/Thailand/CU-1101/2008 | 383 | HQ214396/A/Kisumu/7602/2008 |
| 317 | EU716431/2007731365A/Uruguay/716/2007 | 384 | HQ214400/A/Kisumu/7609/2008 |
| 318 | EU716457/2008707598A/Wisconsin/02/2008 | 385 | HQ214402/A/Kisumu/7614/2008 |
| 319 | EU779505/2008706714A/Tennessee/02/2008 | 386 | JF327388/A/Denmark/22/2011 |
| 320 | EU779509/2008706789A/Minnesota/04/2008 | 387 | JF327389/A/Denmark/23/2011 |
| 321 | EU779513/2008707289A/Memphis/30/2008 | 388 | JF340082/A/Vienna/25/2007 |
| 322 | EU779523/2008708313A/Virginia/02/2008 | 389 | JN256789/A/Hong Kong/H090-763-V1(0)/2009 |
| 323 | EU779531/2008740857A/Missouri/01/2008 | 390 | JN256790/A/Hong Kong/H090-763-V2(3)/2009 |
| 324 | EU791306/A/Ulaanbaatar/1380/2007 | 391 | JN256795/A/Hong Kong/H090-781-V1(0)/2009 |
| 325 | EU852006/2008741850A/Texas/06/2008 | 392 | JN790517/A/Gunma/10G041/2011 |
| 326 | EU852008/2008742684A/Iowa/01/2008 | 393 | JN790518/A/Gunma/10G045/2011 |
| 327 | EU885537/A/Minnesota/07/2008 | 394 | JN790534/A/Hokkaido/10H079/2011 |
| 328 | FJ179351/A/Michigan/01/2008 | 395 | JQ988028/A/Penza/55/2008 |
| 329 | FJ183469/A/Thueringen/2202/2008 | 396 | JQ988034/A/Moscow/02/2012 |
| 330 | FJ229853/A/Myanmar/M109/2007 |  |  |
| 331 | FJ229854/A/Myanmar/M154/2007 |  |  |
| 332 | FJ229855/A/Myanmar/M158/2007 |  |  |
| 333 | FJ769870/A/Tehran/379/2007 |  |  |
| 334 | FJ769873/A/Tehran/487/2007 |  |  |
| 335 | FJ769874/A/Esfahan/501/2007 |  |  |
| 336 | FJ769876/A/Kermanshah/580/2007 |  |  |
| 337 | FJ769878/A/Tehran/631/2007 |  |  |
| 338 | FJ769879/A/Kermanshah/687/2007 |  |  |
| 339 | FJ805592/A/Taiwan/754/2006 |  |  |
| 340 | FJ805593/A/Taiwan/758/2006 |  |  |
| 341 | FJ805602/A/Taiwan/449/2007 |  |  |
| 342 | FJ865343/A/Cambodia/R453/2007 |  |  |
| 343 | FJ912986/A/Thailand/CU370/2008 |  |  |
| 344 | FJ966234/A/Myanmar/M248/2007 |  |  |
| 345 | GQ293084/A/Philippines/16/2009 |  |  |
| 346 | GQ293086/A/Philippines/5/2009 |  |  |
| 347 | GQ293088/A/Singapore/39/2009 |  |  |
