# Supplementary material for: Evolutionary study and phylodynamic pattern of human influenza A/H3N2 virus in Indonesia from 2008 to 2010
Source: PLoS One. 2018 Aug 1;13(8):e0201427. doi: 10.1371/journal.pone.0201427 (PMC6070282; doi:10.1371/journal.pone.0201427)
Supplement: S3 Table — (DOCX) [file pone.0201427.s003.docx]

**S2 Table. Positively selected sites within Indonesian HA gene**

| **Codon** | **Mean probability** | **Omega (*d*_N_/*d*_S_)** | **Function*** | **Reference** |
| --- | --- | --- | --- | --- |
| **HA1 domain** |  |  |  |  |
| 204 | 5.041930000233612e-01 | 2.087028227288444e+00 |  |  |
| 190 | 5.317865195363795e-01 | 2.247377470824323e+00 | B, R | (Suzuki, 2006; Wiley *et al.*, 1981) |
| 34 | 6.084863079213839e-01 | 2.200695098838257e+00 |  |  |
| 6 | 7.532720957080851e-01 | 2.629366614958455e+00 |  |  |
| 38 | 7.532720957080851e-01 | 2.629366614958455e+00 | G | (Bragstad *et al*., 2008) |
| 81 | 7.532720957080851e-01 | 2.629366614958455e+00 | E | (Suzuki, 2006; Wiley *et al*., 1981) |
| 126 | 7.532720957080851e-01 | 2.629366614958455e+00 | A, G | (Bragstad *et al*., 2008; Suzuki, 2006; Wiley *et al*., 1981) |
| 152 | 7.532720957080851e-01 | 2.629366614958455e+00 | A | (Suzuki, 2006; Wiley *et al*., 1981) |
| 171 | 7.532720957080851e-01 | 2.629366614958455e+00 | D | (Suzuki, 2006; Wiley *et al*., 1981) |
| 225 | 7.532720957080851e-01 | 2.629366614958455e+00 | R | (Bragstad *et al*., 2008) |
| 250 | 7.532720957080851e-01 | 2.629366614958455e+00 |  |  |
| 290 | 7.532720957080851e-01 | 2.629366614958455e+00 |  |  |
| 296 | 7.532720957080851e-01 | 2.629366614958455e+00 |  |  |
| 317 | 7.565518360270783e-01 | 2.916641383751844e+00 | TCE | (Carmichael *et al.*, 1997; O'Sullivan *et al.*, 1991) |
| 301 | 8.328147755364111e-01 | 2.987185068563894e+00 |  |  |
| 177 | 9.466275860792027e-01 | 3.170032068611541e+00 | D | (Suzuki, 2006; Wiley *et al*., 1981) |
| 25 | 9.999999996045011e-01 | 3.306180196837575e+00 |  |  |
| 51 | 9.999999996045011e-01 | 3.306180196837575e+00 | C | (Suzuki, 2006; Wiley *et al*., 1981) |
| 67 | 9.999999996045011e-01 | 3.306180196837575e+00 | E | (Suzuki, 2006; Wiley *et al*., 1981) |
| 236 | 9.999999996045011e-01 | 3.306180196837575e+00 |  |  |
| 267 | 9.999999996045011e-01 | 3.306180196837575e+00 |  |  |
| **HA2 domain** |  |  |  |  |
| 501 | 5.275494752765573e-01 | 2.356617980115568e+00 |  |  |
| 402 | 5.708971124224911e-01 | 2.302436119073331e+00 |  |  |
| 437 | 6.218201449766501e-01 | 2.736418064659888e+00 | TCE | (Gianfrani *et al.*, 2000; Suzuki, 2006) |
| 521 | 6.473198519207873e-01 | 2.473732440761711e+00 |  |  |
| 366 | 6.489721210357732e-01 | 2.665348239245676e+00 |  |  |
| 500 | 7.346192126909951e-01 | 2.600937848357742e+00 |  |  |
| 341 | 7.532720957080851e-01 | 2.629366614958455e+00 |  |  |
| 357 | 7.532720957080851e-01 | 2.629366614958455e+00 |  |  |
| 378 | 7.532720957080851e-01 | 2.629366614958455e+00 |  |  |
| 382 | 7.532720957080851e-01 | 2.629366614958455e+00 |  |  |
| 458 | 7.532720957080851e-01 | 2.629366614958455e+00 |  |  |
| 464 | 7.532720957080851e-01 | 2.629366614958455e+00 |  |  |
| 475 | 7.532720957080851e-01 | 2.629366614958455e+00 |  |  |
| 416 | 7.617427651994770e-01 | 2.776657708227565e+00 |  |  |
| 348 | 7.629665780418503e-01 | 2.844986866732876e+00 | TCE | (Gianfrani *et al*., 2000; Suzuki, 2006) |
| 522 | 8.135631274918875e-01 | 2.840104542645511e+00 |  |  |
| 427 | 8.213641754356588e-01 | 2.944600535983943e+00 |  |  |
| 468 | 8.320763369766067e-01 | 2.885639371758329e+00 |  |  |
| 497 | 8.645712361806249e-01 | 2.969467039137479e+00 |  |  |
| 331 | 9.999999996045011e-01 | 3.306180196837575e+00 |  |  |
| 339 | 9.999999996045011e-01 | 3.306180196837575e+00 |  |  |
| 469 | 9.999999996045011e-01 | 3.306180196837575e+00 |  |  |
| 478 | 9.999999996045011e-01 | 3.306180196837575e+00 |  |  |
|  |  |  |  |  |
| Overall omega(-) | 1.363600060094881e-01 |  |  |  |
| Overall omega(N) | 1.000000000000000e+00 |  |  |  |
| Overall omega(+) | 3.306180197921325e+00 |  |  |  |

* Function in capital letters were described as follow: A to E refers to antigenic sites (B-cell epitope) A to E; R refers to receptor binding site; G refers to Glycosylation site; TCE refers to T-cell epitope.
