# Supplementary material for: Evolutionary study and phylodynamic pattern of human influenza A/H3N2 virus in Indonesia from 2008 to 2010
Source: PLoS One. 2018 Aug 1;13(8):e0201427. doi: 10.1371/journal.pone.0201427 (PMC6070282; doi:10.1371/journal.pone.0201427)
Supplement: S4 Table — (DOCX) [file pone.0201427.s004.docx]

**S3 Table. Positively selected sites within Indonesian NA gene**

| **Codon** | **Mean probability** | **Omega (*d*_N_/*d*_S_)** | **Function*** | **Reference** |
| --- | --- | --- | --- | --- |
| 233 | 5.137560076965452e-01 | 1.983485572940307e+00 | TCE | (Suzuki, 2006; Wedemeyer *et al.*, 2001) |
| 366 | 5.137560076965452e-01 | 1.983485572940307e+00 | Head, C | (Air *et al.*, 1985; Suzuki, 2006) |
| 227 | 5.143475700283334e-01 | 1.986212774312309e+00 |  |  |
| 101 | 5.148307016806467e-01 | 1.843231566978655e+00 |  |  |
| 180 | 5.182832998541359e-01 | 1.962762326435674e+00 |  |  |
| 204 | 5.182832998541359e-01 | 1.962762326435674e+00 |  |  |
| 311 | 5.182832998541359e-01 | 1.962762326435674e+00 |  |  |
| 331 | 5.182832998541359e-01 | 1.962762326435674e+00 | Head, C | (Air *et al*., 1985; Colman *et al*., 1983; Suzuki, 2006) |
| 25 | 5.220307571774705e-01 | 1.853404902194410e+00 |  |  |
| 39 | 5.220307571774705e-01 | 1.853404902194410e+00 |  |  |
| 49 | 5.220307571774705e-01 | 1.853404902194410e+00 |  |  |
| 91 | 5.220307571774705e-01 | 1.853404902194410e+00 | TCE | (Gianfrani *et al*., 2000; Suzuki, 2006) |
| 131 | 5.220307571774705e-01 | 1.853404902194410e+00 |  |  |
| 173 | 5.220307571774705e-01 | 1.853404902194410e+00 |  |  |
| 395 | 5.220307571774705e-01 | 1.853404902194410e+00 |  |  |
| 263 | 5.406630007733880e-01 | 2.111420889983312e+00 |  |  |
| 300 | 5.510608677682292e-01 | 2.127680349955779e+00 |  |  |
| 428 | 5.510608677682292e-01 | 2.127680349955779e+00 |  |  |
| 112 | 5.555193627577607e-01 | 2.091730040308919e+00 |  |  |
| 196 | 5.555193627577607e-01 | 2.091730040308919e+00 |  |  |
| 461 | 5.555193627577607e-01 | 2.091730040308919e+00 |  |  |
| 128 | 5.566614716485312e-01 | 2.062375709736290e+00 |  |  |
| 267 | 5.567851903101624e-01 | 1.869469588642716e+00 | Head | (Westgeest *et al*., 2012) |
| 12 | 5.578965482087141e-01 | 1.993118018190019e+00 |  |  |
| 14 | 5.578965482087141e-01 | 1.993118018190019e+00 |  |  |
| 407 | 5.578965482087141e-01 | 1.993118018190019e+00 |  |  |
| 411 | 5.578965482087141e-01 | 1.993118018190019e+00 |  |  |
| 93 | 5.593440702703316e-01 | 2.209213287543527e+00 | TCE | (Gianfrani *et al*., 2000; Suzuki, 2006) |
| 103 | 5.593440702703316e-01 | 2.209213287543527e+00 |  |  |
| 113 | 5.593440702703316e-01 | 2.209213287543527e+00 |  |  |
| 293 | 5.593440702703316e-01 | 2.209213287543527e+00 |  |  |
| 324 | 5.593440702703316e-01 | 2.209213287543527e+00 |  |  |
| 330 | 5.593440702703316e-01 | 2.209213287543527e+00 | Head, C | (Air *et al*., 1985; Colman *et al*., 1983; Suzuki, 2006) |
| 359 | 5.593440702703316e-01 | 2.209213287543527e+00 | Head, C | (Air *et al*., 1985; Suzuki, 2006) |
| 463 | 5.593440702703316e-01 | 2.209213287543527e+00 |  |  |
| 11 | 5.606580902315564e-01 | 1.963616329818417e+00 |  |  |
| 427 | 5.665263806068189e-01 | 1.963446235187763e+00 |  |  |
| 266 | 5.665454139037612e-01 | 2.133519561997554e+00 |  |  |
| 338 | 5.706424395046430e-01 | 2.180690162819086e+00 | Head, C | (Air *et al*., 1985; Colman *et al*., 1983; Suzuki, 2006) |
| 347 | 5.731407014774133e-01 | 2.137490448016152e+00 | Head, C | (Air *et al*., 1985; Colman *et al*., 1983; Suzuki, 2006) |
| 215 | 5.736618992116944e-01 | 2.131502474374813e+00 |  |  |
| 260 | 5.782310101452995e-01 | 2.183969574568783e+00 |  |  |
| 195 | 5.795683339587581e-01 | 2.004297403936377e+00 |  |  |
| 125 | 5.891654349794604e-01 | 2.224791696990237e+00 |  |  |
| 119 | 5.953579720047701e-01 | 2.061381614952171e+00 |  |  |
| 202 | 6.094548252889576e-01 | 2.232692831462579e+00 |  |  |
| 399 | 6.186864600434914e-01 | 2.184653313483017e+00 | Head, A | (Air *et al*., 1985; Colman *et al*., 1983; Suzuki, 2006) |
| 436 | 6.201201122917946e-01 | 2.191802081671328e+00 |  |  |
| 197 | 6.201794730078846e-01 | 2.266126042936555e+00 | Head, B | (Air *et al*., 1985; Colman *et al*., 1983; Suzuki, 2006) |
| 335 | 6.308705527980156e-01 | 2.262335508807741e+00 |  |  |
| 69 | 6.376738442373983e-01 | 2.436305560623199e+00 |  |  |
| 71 | 6.376738442373983e-01 | 2.436305560623199e+00 |  |  |
| 153 | 6.376738442373983e-01 | 2.436305560623199e+00 |  | (Colman *et al*., 1983) |
| 171 | 6.376738442373983e-01 | 2.436305560623199e+00 |  |  |
| 225 | 6.376738442373983e-01 | 2.436305560623199e+00 |  |  |
| 376 | 6.376738442373983e-01 | 2.436305560623199e+00 |  |  |
| 449 | 6.376738442373983e-01 | 2.436305560623199e+00 |  |  |
| 99 | 6.436862996688251e-01 | 2.429054958357694e+00 | TCE | (Gianfrani *et al*., 2000; Suzuki, 2006) |
| 126 | 6.436862996688251e-01 | 2.429054958357694e+00 |  |  |
| 154 | 6.436862996688251e-01 | 2.429054958357694e+00 |  |  |
| 282 | 6.436862996688251e-01 | 2.429054958357694e+00 |  |  |
| 285 | 6.436862996688251e-01 | 2.429054958357694e+00 |  |  |
| 459 | 6.436862996688251e-01 | 2.429054958357694e+00 |  |  |
| 62 | 6.449232342658998e-01 | 2.427679466924228e+00 |  |  |
| 194 | 6.458504938864665e-01 | 2.424718898448805e+00 |  |  |
| 9 | 6.515870719867618e-01 | 2.462974763285618e+00 |  |  |
| 275 | 6.606737350776789e-01 | 2.395649012429633e+00 |  |  |
| 287 | 6.606737350776789e-01 | 2.395649012429633e+00 |  |  |
| 290 | 6.606737350776789e-01 | 2.395649012429633e+00 |  |  |
| 379 | 6.606737350776789e-01 | 2.395649012429633e+00 |  |  |
| 367 | 6.675749437734135e-01 | 2.428963331164264e+00 | Head, C | (Air *et al*., 1985; Colman *et al*., 1983; Suzuki, 2006) |
| 368 | 6.686109703172277e-01 | 2.298427592119998e+00 | Head, C | (Air *et al*., 1985; Colman *et al*., 1983; Suzuki, 2006) |
| 199 | 6.738028259622179e-01 | 2.481380034433292e+00 | Head, B | (Air *et al*., 1985; Colman *et al*., 1983; Suzuki, 2006) |
| 430 | 6.798821285840070e-01 | 2.328489076406924e+00 |  |  |
| 90 | 6.838574369537290e-01 | 2.540117865089193e+00 | TCE | (Gianfrani *et al*., 2000; Suzuki, 2006) |
| 70 | 6.852816426119458e-01 | 2.309011552647128e+00 |  |  |
| 370 | 6.919491309607952e-01 | 2.462118442380305e+00 | Head, C | (Air *et al*., 1985; Colman *et al*., 1983; Suzuki, 2006) |
| 107 | 7.026306979812448e-01 | 2.560716662378293e+00 |  |  |
| 198 | 7.060287171570819e-01 | 2.546492112552653e+00 | Head, B | (Air *et al*., 1985; Colman *et al*., 1983; Suzuki, 2006) |
| 318 | 7.241047493605697e-01 | 2.572400828006898e+00 |  |  |
| 355 | 7.256260513993233e-01 | 2.610220374171281e+00 |  |  |
| 298 | 7.283121309780481e-01 | 2.730321950970631e+00 |  |  |
| 390 | 7.375284059665180e-01 | 2.684344095077348e+00 | Head, A | (Air *et al*., 1985; Colman *et al*., 1983; Suzuki, 2006) |
| 308 | 7.436011633663334e-01 | 2.656178935178170e+00 | Head | (Westgeest *et al*., 2012) |
| 240 | 7.813732648238494e-01 | 2.540575860214457e+00 | TCE | (Suzuki, 2006; Wedemeyer *et al*., 2001) |
| 464 | 7.857539105346398e-01 | 2.703325285949555e+00 |  |  |
| 323 | 8.046626387748192e-01 | 2.764712064642135e+00 |  |  |
| 89 | 9.916921123622465e-01 | 3.015781970697503e+00 |  |  |
|  |  |  |  |  |
| Overall omega(-) | 4.461241185348387e-01 |  |  |  |
| Overall omega(N) | 1.000000000000000e+00 |  |  |  |
| Overall omega(+) | 3.032477207894587e+00 |  |  |  |

* Function in capital letters were described as follow: A to C refers to antigenic sites (B-cell epitope) A to C; TCE refers to T-cell epitope
