# Supplementary figures and images for: Evolutionary study and phylodynamic pattern of human influenza A/H3N2 virus in Indonesia from 2008 to 2010
Source: PLoS One. 2018 Aug 1;13(8):e0201427. doi: 10.1371/journal.pone.0201427 (PMC6070282; doi:10.1371/journal.pone.0201427)

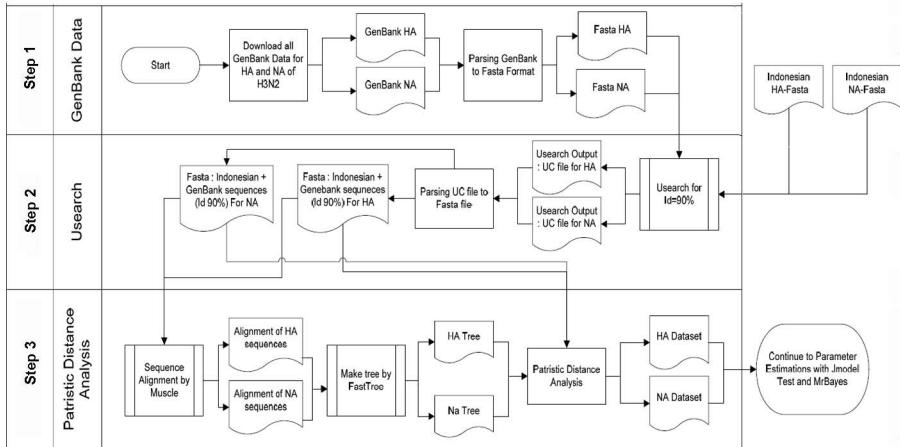

Supplement: S1 Fig — (PDF) [file pone.0201427.s005.pdf]

(a)

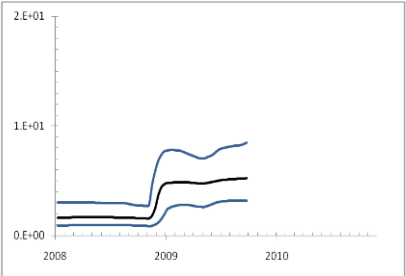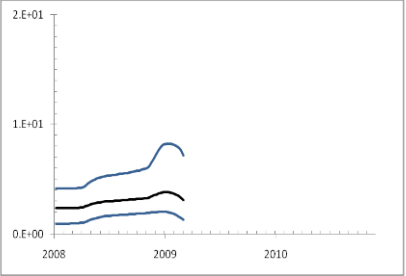

(b)

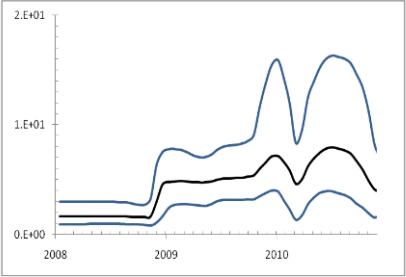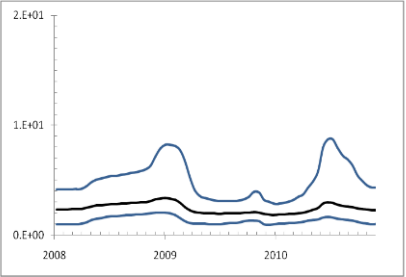

(c)

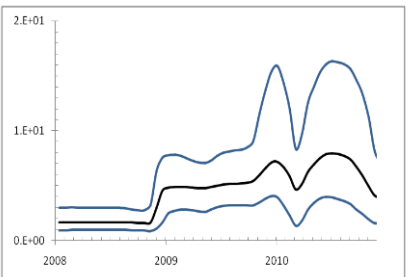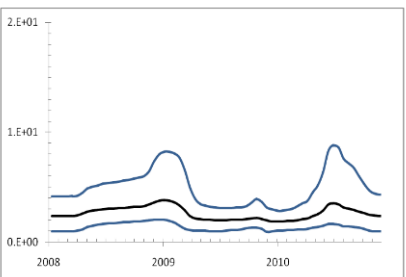

(d)

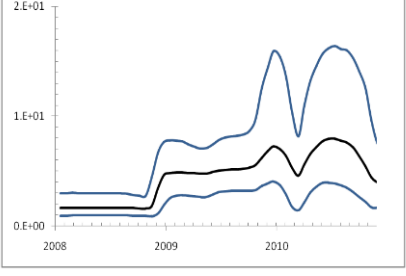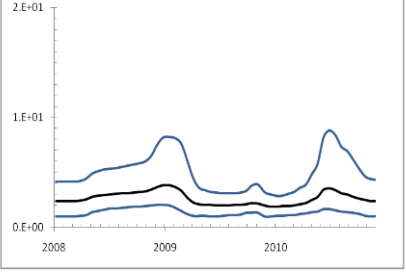

(e)

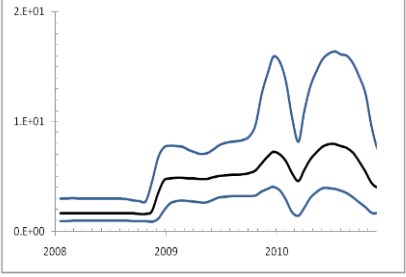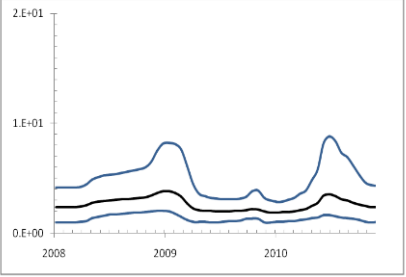

(f)

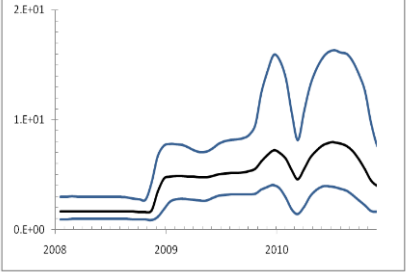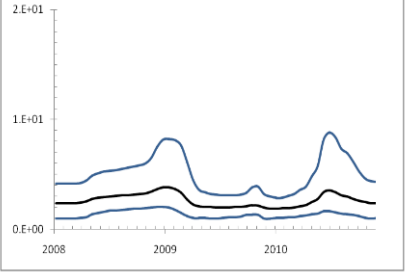

(g)

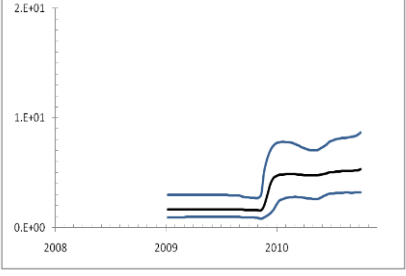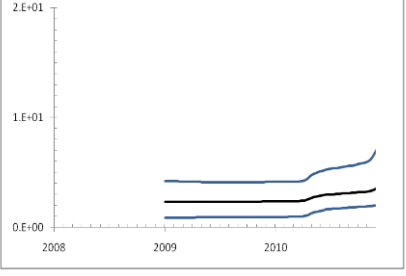

Supplement: S2 Fig — Datasets were divided based on the samples origin as follow: Balikpapan (a), Banjarmasin (b), Batam, Aceh and Medan (c), Java and Lampung (d), Jayapura (e), Makasar (f), and Merauke (g). The Bayesian skyline plot at the left panel was genererated from HA gene while at the right panel from NA gene. The x-axis represents the time (mm/yy) and the y-axis represents a measure of relative genetic diveristy (Net, where the Ne is the effective population size and t the generation time from infected host to infected host). The black line represents the mean value while the 95% confidence limits shown in blue line. (PDF) [file pone.0201427.s006.pdf]
