# Supplementary material for: Evolutionary study and phylodynamic pattern of human influenza A/H3N2 virus in Indonesia from 2008 to 2010
Source: PLoS One. 2018 Aug 1;13(8):e0201427. doi: 10.1371/journal.pone.0201427 (PMC6070282; doi:10.1371/journal.pone.0201427)
Supplement: S3 Fig — The Indoneisan sequences were denoted with red labels, while sequences from Southern, Northern Hemispheres, and Tropics were denoted with black labels. Banch color scheme, scale and symbols are similar as those in Fig 2. (PDF) [file pone.0201427.s007.pdf]

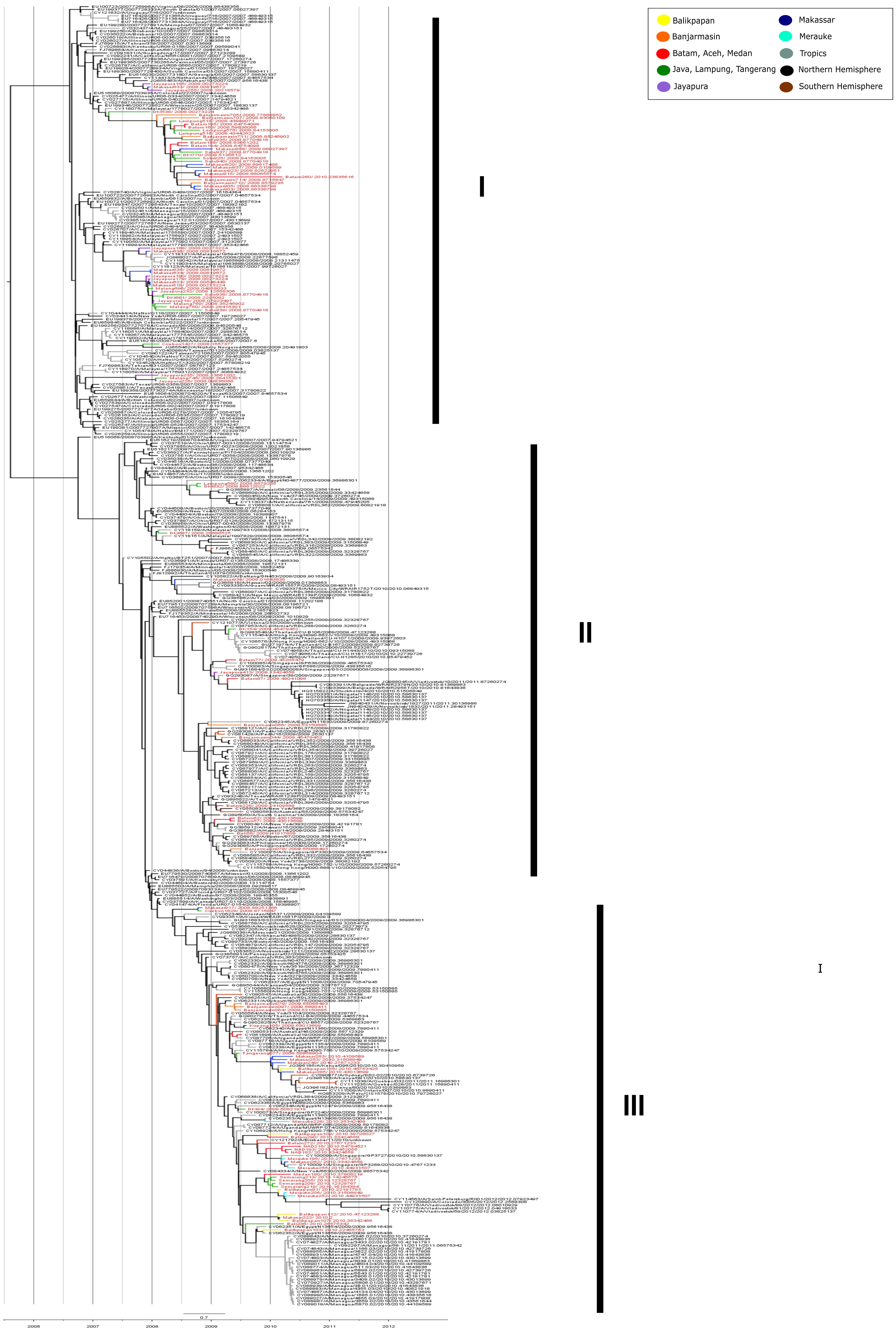

Balikpapan
Banjarmasin
Batam, Aceh, Medan
Java, Lampung, Tangerang
Jayapura
Makassar
Merauke
Tropics
Northern Hemisphere
Southern Hemisphere
I
II
III
0.7
2006
2007
2008
2009
2010
2011
2012
